# Meiotic prophase I disruption as a strategy for nonhormonal male contraception using small-molecule inhibitor JQ1

**DOI:** 10.1073/pnas.2517498123

**Published:** 2026-04-07

**Authors:** Stephanie Tanis, Leah E. Simon, Adriana K. Alexander, Tegan S. Horan, Maria de las Mercedes Carro, Samantha Jane Bonnett, Audrey Xie, Roni Ben-Shlomo, Connor E. Owens, Charles G. Danko, Jelena Lujic, Paula E. Cohen

**Affiliations:** ^a^Department of Biomedical Sciences and Cornell Reproductive Sciences Center, College of Veterinary Medicine, Cornell University, Ithaca, NY 14853; ^b^Baker Institute for Animal Health, College of Veterinary Medicine, Cornell University, Ithaca, NY 14850

**Keywords:** transcription, mouse, spermatogenesis, male contraception, meiosis

## Abstract

Developing safe, reversible, and nonhormonal male contraceptives has been hindered by the lack of defined biological windows that can be transiently interrupted without compromising long-term fertility. Here, we tested whether meiotic prophase I can serve as such a window by pharmacologically inhibiting the testis-specific chromatin reader BRDT using the small-molecule bromodomain inhibitor (+)-JQ1 as proof-of-principle. Short-term JQ1 administration (3 wk) selectively disrupted the pachytene transcriptional program, depleted postmeiotic germ cells, and induced a reversible arrest in spermatogenesis. Upon drug withdrawal, prophase I cytological markers normalized within 6 wk, accompanied by restoration of testis architecture and germ-cell composition. Crossover metrics and transcriptional programs recovered more gradually, reaching full normalization by 30 wk alongside complete restoration of fertility and fecundity. These results demonstrate that meiotic prophase I can be transiently inhibited to suppress spermatogenesis reversibly without inducing lasting genomic or reproductive defects, defining a stage-specific framework for the rational design of nonhormonal male contraceptives.

## Significance

Few reversible male contraceptives have advanced toward clinical translation, largely because the optimal biological stage for safe intervention remains undefined. Meiosis represents a natural checkpoint in sperm production where transient inhibition could achieve precise and reversible fertility control. Using the small-molecule BRDT inhibitor (+)-JQ1, we demonstrate that brief suppression of meiotic prophase I halts spermatogenesis yet permits complete recovery of germ-cell differentiation, recombination fidelity, and fertility after withdrawal. While acknowledging the need for robust future safety assessments of any candidate drugs, these studies provide a blueprint for developing new contraceptive approaches that act safely and selectively within the germline.

Unintended pregnancies account for nearly 44% of all pregnancies worldwide, underscoring the need for expanded contraceptive options that engage men (1). Existing approaches are largely female-centered or permanent, such as vasectomy, leaving few reversible male options (2). Developing nonhormonal methods that transiently prevent sperm production would help close this gap and promote shared reproductive responsibility.

Spermatogenesis, the process by which diploid spermatogonia divide and differentiate into mature spermatozoa (3), proceeds through three major stages: i) the spermatogonial phase, during which spermatogonial stem cells (SSCs) self-renew, proliferate, and differentiate; ii) the meiotic phase, in which primary spermatocytes undergo homologous pairing, recombination, and two reductional divisions to form haploid spermatids; and iii) the spermiogenic phase, where spermatids undergo transcriptional silencing and structural remodeling to form spermatozoa. By transiently disrupting key regulators at defined stages, it may be possible to halt sperm production reversibly without compromising the SSC pool (4). Advances in genomics and single-cell transcriptomics now enable high-resolution mapping of these stages, revealing stage-specific molecular targets (5, 6).

A central challenge in identifying the appropriate target is selecting the optimal developmental window. Early disruption of spermatogonia risks damaging the SSC niche, whereas postmeiotic interventions risk residual fertilization. Meiosis, in which diploid precursors divide to form haploid gametes, offers a potential balance, as it combines distinct cellular hallmarks of homolog pairing, synapsis, and recombination (7) with a tightly regulated “pachytene transcriptional burst” that transitions the meiotic to spermiogenic program (8). Temporarily perturbing this stage could therefore block sperm formation with temporal precision while preserving regenerative capacity.

The testis-specific bromodomain protein BRDT is a key regulator of pachytene transcription (8, 9). Matzuk et al. showed that (+)-JQ1 (JQ1), a small-molecule bromodomain inhibitor, induces reversible infertility in mice by inhibiting BRDT (10). Because every developing sperm passes through this stage, transient BRDT inhibition provides a tractable model to test whether meiotic prophase I can be pharmacologically targeted to achieve reversible contraception without lasting molecular or genomic defects.

Here, we extended the foundational studies of Matzuk and colleagues to directly evaluate the reversibility and molecular consequences of transient meiotic inhibition. Using JQ1 as a probe compound, we assessed fertility outcomes, pachytene transcriptional dynamics, and meiotic chromosome architecture through single-cell RNA sequencing, histology, and cytogenetic analyses before and after treatment, as well as in F1 male progeny from treated fathers. These analyses reveal that transient BRDT inhibition disrupts the pachytene transcriptional program yet allows complete recovery of spermatogenesis, recombination fidelity, and fertility after withdrawal. Together, they establish meiotic prophase I as a biologically defined and reversible control point within the spermatogenic program, providing a framework for evaluating future stage-specific contraceptive strategies.

## Results

### Inhibition of BRDT Causes Infertility By Disrupting the Switch From the Meiotic to the Spermiogenic Transcriptome During Spermatogenesis.

**Fig. 1.**
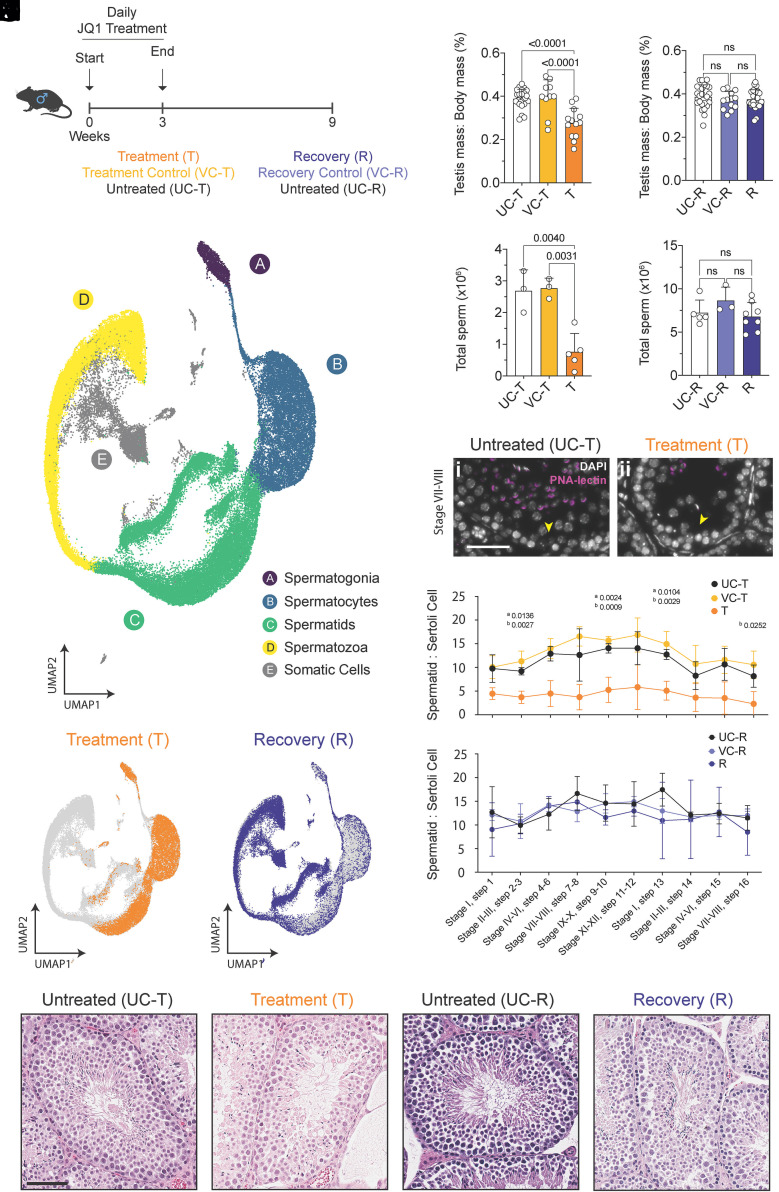
Three-week JQ1 treatment causes postmeiotic cell loss with full recovery after withdrawal. (*A*) Experimental design outlining 3 wk of daily JQ1 injections (treatment, T) or vehicle controls (VC-T) with age-matched untreated controls (UC-T). Recovery was assessed 6 wk after cessation of JQ1 (R) or vehicle (VC-R), alongside untreated recovery controls (UC-R). (*B* and *C*) Testis mass (% of body mass) for (*B*) treatment timepoints (T, n = 8; VC-T, n = 7; UC-T, n = 13 mice) and (*C*) recovery timepoints (R, n = 14; VC-R, n = 7; UC-R, n = 13 mice). Statistical comparisons were performed using one-way ANOVA; exact *P* values are shown. (*D* and *E*) Total epididymal sperm counts (10^6^ sperm per mouse) for (*D*) treatment cohorts (T, n = 5; VC-T, n = 3; UC-T, n = 3 mice) and (*E*) recovery cohorts (R, n = 8; VC-R, n = 3; UC-R, n = 5 mice). Statistical comparisons were performed using one-way ANOVA (*D*) or Kruskal–Wallis test with Dunn’s post hoc test (*E*); exact *P* values are shown. (*F*–*H*) UMAP visualizations of all single-cell RNA-seq libraries across treatment and recovery conditions (*F*), highlighting cell populations captured after treatment (*G)* and recovery (*H*). Each point represents one cell colored by germ-cell type. (*I* and *J*) Representative testicular histology from untreated (UC-T, *I*) and JQ1-treated (T, *J*) males by hematoxylin and eosin (H&E) staining. (*K*) Immunofluorescence staining of stage VII–VIII seminiferous tubules using PNA-lectin (magenta) and DAPI (grayscale) in UC-T and T testes. Yellow arrows indicate Sertoli cells. (Scale bar, 50 µm.) (*L* and *M*) Quantification of spermatid-to-Sertoli cell ratios per seminiferous tubule for (*L*) UC-T, VC-T, and T males and (*M*) VC-R, R, and UC-R males (n = 3 mice per group). Statistical comparisons were performed using one-way ANOVA; superscripts denote statistical comparisons (a = T vs UC-T, b = T vs VC-T). Exact *P* values are shown. (*N* and *O*) Representative testicular H&E staining from untreated recovery (UC-R, *N*) and recovery (R, *O*) males showing full restoration of spermatogenesis. (Scale bar, 100 µm.) Data are shown as mean ± SD throughout.

Although JQ1 is known to block spermatogenesis in a reversible fashion, its precise effects on the meiotic transcriptional program and its recovery remain uncharacterized. To explore this, we recapitulated the treatment regime of Matzuk et al. (10), subjecting adult DBA/2J males to daily intraperitoneal injections of JQ1 (50 mg/kg) or vehicle for 3 wk (11). Animals were divided into treatment and vehicle cohorts, each analyzed either immediately after dosing (treatment, T; vehicle control-treatment, VC-T) or 6 wk after JQ1 withdrawal (recovery, R; vehicle control-recovery, VC-R); age-matched untreated males served as baseline controls at both treatment (UC-T) and recovery (UC-R) timepoints (Fig. 1*A*). Testis mass and sperm counts were measured, with JQ1 significantly reducing both parameters relative to controls (Fig. 1 *B* and *D*), while withdrawal of JQ1 for 6 wk restored these values returned to baseline (Fig. 1 *C* and *E*).

We next characterized the genetic basis of this reversible phenotype. Single-cell RNA sequencing (scRNA-seq) of testicular suspensions from treatment, recovery, and control males captured 69,262 high-quality cells spanning all major germ and somatic populations (Fig. 1*F* and *SI Appendix*, Fig. S2). Clustering and UMAP visualization revealed the full continuum of spermatogenic stages, confirming comprehensive sampling (*SI Appendix*, Figs. S3 and S4). In JQ1-treated testes, germ cells progressed normally through meiosis to the haploid spermatid stage but failed to produce mature spermatozoa (Fig. 1*G*), consistent with the known contraceptive effect of BRDT inhibition. Histological analyses supported this finding: hematoxylin–eosin staining showed a depletion of luminal spermatozoa, and PNA-lectin labeling revealed a reduction across spermatid steps 1 to 16 in treated males relative to controls (Fig. 1 *I*–*L*). Mild increases in TUNEL-positive cells indicated limited apoptosis (*SI Appendix*, Fig. S5 *A* and *B*) rather than widespread damage.

To confirm testis specificity of the JQ1 effect, we performed parallel RNA-seq and histological analyses of kidney and liver. JQ1 induced only minimal, reversible transcriptional changes in nongonadal tissues and preserved normal histological architecture in both organs (*SI Appendix*, Fig. S6 *B*–*D*). Within the testis, we observed no impact on Sertoli cell abundance or function, demonstrating largely germ-cell-restricted activity and complete recovery following withdrawal (*SI Appendix*, Fig. S6 *F*–*H*). Equivalent RNA-seq analyses performed at the recovery timepoint showed full normalization of tissue morphology and gene expression, confirming that the minimal off-target effects were transient (*SI Appendix*, Fig. S6 *B*, *C*, and *E*). By 6 wk postdrug cessation, germ-cell composition and seminiferous architecture were indistinguishable from controls. Recovery testes displayed the full complement of spermatogenic stages in both scRNA-seq and histology (Fig. 1 *H*–*M*), with normal apoptotic indices (*SI Appendix*, Fig. S5 *C* and *D*).

### BRDT Inhibition Disrupts the Prophase I Progression and the Pachytene Transcriptional Switch Leading to Haploid Germ Cell Loss.

**Fig. 2.**
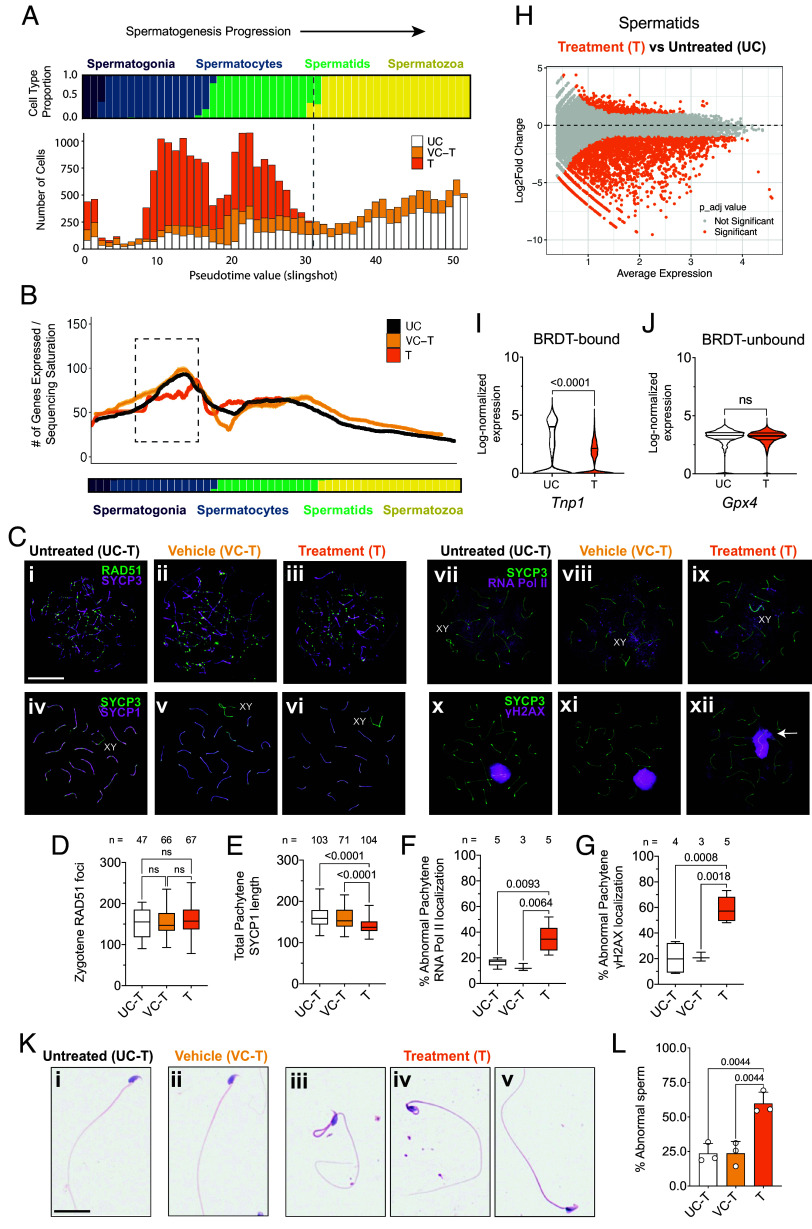
Transient BRDT inhibition disrupts pachytene transcription and drives haploid cell loss. (*A*) Pseudotime ordering of single-cell transcriptomes showing spermatogenic progression (*Top*) and cell density by condition (*Bottom*). The dashed line indicates the spermatid-to-spermatozoa transition, where JQ1-treated (T) testes show cell loss. (*B*) Transcriptional activity (number of genes expressed per cell) plotted along pseudotime for untreated (UC) (black), vehicle (VC-T) (yellow), and JQ1-treated (T) (orange) testes. The dashed box highlights attenuation of the pachytene transcriptional burst in treated samples. (*C*) Representative chromosome spreads from spermatocytes labeled for markers of recombination and transcription: (*i*–*iii*) RAD51 and SYCP3 during zygonema; (*iv*–*vi*) SYCP1 and SYCP3 during pachynema; (*vii*–*ix*) SYCP3 and RNA polymerase II at pachynema; (*x*–*xii*) SYCP3 and γH2AX at pachynema. (Scale bar, 20 µm.) (*D*–*G*) Quantification of meiotic phenotypes: (*D*) RAD51 foci per zygotene nucleus (n = 47 to 67 nuclei, 3 mice per group), (*E*) total SYCP1 length per pachytene nucleus (n = 71 to 104 nuclei, 3 to 5 mice per group), (*F*) percentage of pachytene nuclei with autosomal RNA polymerase II localization (minimum 30 nuclei averaged across n = 3 to 5 mice), (*G*) percentage with γH2AX signal extending beyond the sex body (minimum 30 nuclei averaged across n = 3 to 5 mice). Boxplots show median and interquartile range. Statistical comparisons were performed using one-way ANOVA (*F* and *G*) or Kruskal–Wallis test with Dunn’s post hoc test (*D* and *E*); exact *P* values are shown. (*H*) Differential expression (DE) analysis of pseudobulked spermatids comparing treated (T) and untreated (UC) testes. Each point on MA plot represents one gene; significantly altered genes (adjusted *P* < 0.05, |log_2_fold change| > 1) are shown in orange. (*I* and *J*) Examples of spermatid genes with differential or unchanged expression between conditions. *Tnp1* (*I*) is BRDT-bound and downregulated in treated testes; *Gpx4* (*J*), whose promoter lacks BRDT binding, is unaffected. Violin plots show log-normalized expression; significance determined by Student’s *t* test (ns = not significant). (*K*) Representative H&E-stained epididymal sperm from untreated (UC-T) (*i*), vehicle (VC-T) (*ii*), and treated (T) (*iii*–*v*) males, showing abnormal head and flagellar morphology after JQ1 treatment. (Scale bar, 20 µm.) (*L*) Percentage of abnormal sperm per male (n = 3 to 5 mice per group). Data are shown as mean ± SD. Statistical comparisons were performed using one-way ANOVA; exact *P* values are shown.

We next sought to define the specific stage and mechanism of spermatogenic failure. All single-cell transcriptomes were ordered along pseudotime using Slingshot (12) (*SI Appendix*, Fig. S7). Cell density mapping across 51 evenly spaced bins revealed a marked loss at the spermatid-to-spermatozoa transition (Fig. 2*A*; dashed line), matching histological reduction observed in haploid cells (Fig. 1). Analysis of transcriptional dynamics clarified the origin of this cell loss. In control testes, transcription followed a characteristic biphasic pattern of spermatogenesis: a major burst in spermatocytes followed by a smaller wave in spermatids that wanes during sperm maturation (13) (Fig. 2*B*). JQ1 treatment selectively attenuated the pachytene transcriptional burst (Fig. 2*B*; dashed rectangle), indicating loss of coordinated gene activation at mid-prophase I. Because pachytene transcription largely establishes the mRNA pool required for later spermiogenic remodeling (14), its attenuation predicts downstream haploid loss. BRDT remained detectable in pachytene and postmeiotic germ cells across all mice from treatment timepoint (*SI Appendix*, Fig. S6*A*), confirming that the phenotype reflects functional inhibition rather than protein loss (10).

Differential expression (DE) analysis on pseudobulked spermatids revealed 3,195 genes that were altered between treated (T) and untreated (UC) testes (Fig. 2*H*), including key chromatin-remodeling genes during meiosis such as *Smarca5* (13) and sperm-structural genes such as *Tnp1/2* (15) and *Prm1/2* (16) (Fig. 2*I*). Integration with published BRDT ChIP-seq data (11) identified 46 differentially expressed genes in spermatids and 16 in spermatocytes whose promoters are bound by BRDT (*SI Appendix*, Tables S2 and S3), suggesting that JQ1 first disrupts a defined set of BRDT targets, which in turn propagate broader downstream transcriptional changes. In contrast, genes whose promoters lack BRDT binding — such as *Gpx4*, which is essential for spermiogenesis (17)—showed no change in expression (Fig. 2*J*), confirming that JQ1 acts specifically through BRDT-occupied loci rather than broadly repressing spermatid transcription.

We next examined the impact of this transcriptional disruption for the regulation of key prophase I events to understanding the molecular underpinnings of the JQ1 inhibition of meiosis. Early recombination events appeared normal following JQ1 treatment, as defined by the normal zygotene appearance of RAD51 foci on meiotic chromosome cores marked with the synaptonemal complex (SC) marker, SYCP3 (7) (Fig. 2 *C*, *i–iii* and *D*). Chromosome synapsis remains intact, as SYCP1, the central element of the SC (7), continues to colocalize with SYCP3, but total SYCP1 tract length is reduced (Fig. 2 *C, iv–vi* and *E*), indicating shortened SCs rather than asynapsis. This coincides with increased RNA polymerase II localization within the sex body (Fig. 2 *C, vii–ix* and *F*) and aberrant γH2AX signal beyond the sex body (Fig. 2 *C*, *x–xii* and *G*), consistent with failed transcriptional regulation and perturbed chromosomal architecture (7). BRDT protein localization coincided with the onset of pachynema (*SI Appendix*, Fig. S9*B*), temporally matching the appearance of these defects (*SI Appendix*, Fig. S9 *A* and *C*).

Together, these results demonstrate that JQ1 disrupts critical features of pachytene progression, including synapsis, chromatin organization, and the transcriptional burst that leads to the onset of the spermiogenic program, all culminating in severe morphological defects in the resulting spermatozoa. These included abnormal head shaping and bent or kinked flagella (Fig. 2 *K*–*L*), features indicative of defective chromatin condensation (18).

### Meiotic and Spermiogenic Programs Fully Recover After Withdrawal of BRDT Inhibition.

**Fig. 3.**
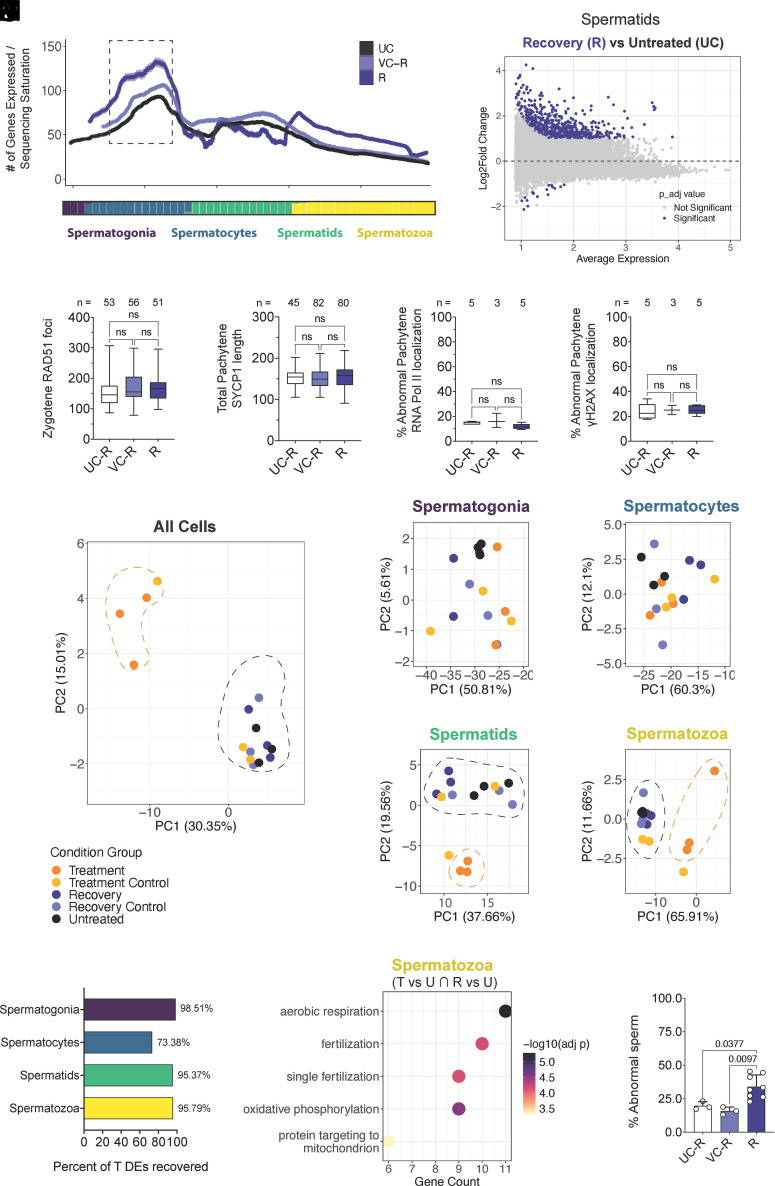
Meiotic and spermiogenic programs fully recover after withdrawal of BRDT inhibition. (*A*) Transcriptional activity plotted along pseudotime for untreated (UC), vehicle (VC-R), and recovery (R) testes. Recovery samples reestablish the canonical pachytene transcriptional burst (dashed box) lost during treatment (Fig. 2*B*). (*B*–*E*) Meiotic chromosome parameters showing full cytological recovery: (*B*) RAD51 foci per zygotene nucleus (n = 51 to 56 nuclei, 3 mice per group), (*C*) total SYCP1 length in pachytene nuclei (n = 45 to 82 nuclei, 3 to 4 mice per group), (*D*) frequency of pachytene nuclei with autosomal RNA polymerase II (minimum 30 nuclei averaged across n = 3 to 5 mice), and (*E*) frequency with γH2AX signal outside the sex body (minimum 30 nuclei averaged across n = 3 to 5 mice). Boxplots show median and interquartile range. Statistical comparisons were performed using one-way ANOVA (*E*–*G*) or Kruskal–Wallis test with Dunn’s post hoc test (*D* and *E*); ns = not significant. (*F*) MA-plot of pseudobulked spermatids comparing recovery (R) versus untreated (UC) testes. Each point represents one gene; purple points indicate significantly differentially expressed genes (adjusted *P* < 0.05, |log_2_fold change| > 1). (*G*–*K*) Principal-component analysis (PCA) of global (*G*) and stage-specific (*H*–*K*) transcriptomes. Treatment (orange) separates from controls, while recovery (dark purple) clusters with untreated samples, indicating stage-specific and reversible effects. (*L*) “Healing” metric showing the proportion of treatment-induced DE genes that returned to baseline after recovery (T vs U ∩ R vs U). Bars show percent recovered DEs for each germ-cell type. (*M*) Gene-ontology enrichment of genes that remained differentially expressed in recovery spermatozoa, highlighting processes related to oxidative phosphorylation, mitochondrial targeting, and fertilization. (*N*) Percentage of abnormal sperm per male (n = 3 to 8 mice per group). Data are shown as mean ± SD. Statistical comparisons were performed using the Kruskal–Wallis test with Dunn’s post hoc test; exact *P* values are shown.

To test JQ1 reversibility, testes from untreated (UC-R), vehicle-treated (VC-R), and recovery (R) males were analyzed 6 wk posttreatment. Progression through prophase I also returned to baseline, with comparable proportions of substages across groups (*SI Appendix*, Fig. S9*D*). Cytological markers of meiosis hallmarks fully normalized: zygotene RAD51 foci, pachytene SYCP1 lengths, and the frequencies of RNA polymerase II sex body encroachment or γH2AX localization outside the sex body were indistinguishable among groups (Fig. 3 *B*–*E*), confirming restoration of both chromosomal organization and transcriptional compartmentalization during prophase I.

Transcriptional activity also recovered after JQ1 cessation, with a return to the canonical pachytene transcriptional burst along pseudotime (Fig. 3*A*; dashed box). Spermatids from R testes displayed elevated transcript diversity and expression relative to controls, consistent with a global increase across germ-cell types (*SI Appendix*, Fig. S10) and reflected in the spermatid-skewed MA profile for R vs. UC (Fig. 3*F*). Principal-component analysis (PCA) supported a stage-specific and reversible effect of JQ1. Across all germ cells, treatment libraries separated from controls, whereas recovery samples clustered closely with untreated and vehicle groups (Fig. 3*G*). Stage-resolved PCAs showed minimal differences among spermatogonia and spermatocytes (Fig. 3 *H* and *I*) but distinct displacement of spermatids and spermatozoa during treatment (Fig. 3 *J* and *K*), consistent with the high variance explained by PC1 and PC2 across these stages (*SI Appendix*, Fig. S11*A*). This separation collapsed in the recovery timepoint, as shown by intersample correlation analysis (*SI Appendix*, Fig. S11*B*), which confirmed tight clustering of recovery samples with controls and complete transcriptional normalization.

To quantify the extent of restoration, we defined a “healing” metric representing the proportion of genes differentially expressed during treatment that returned to baseline after withdrawal (T vs U ∩ R vs U) (Fig. 3*L*). Healing was extensive across germ-cell types, with 98.5% of spermatogonia, 73.4% of spermatocyte, 95.4% of spermatid, and 95.8% of spermatozoa DE genes normalized, indicating that nearly all treatment-associated transcriptional perturbations are reversible. The comparatively lower recovery within spermatocytes mirrors the transient delay in reinstating the pachytene transcriptional burst (Fig. 3*A*), whereas the high recovery in spermatids and spermatozoa underscores reactivation of downstream gene programs once meiosis resumes.

Although spermatozoa exhibited substantial transcriptional healing, a subset of genes remained differentially expressed, enriched for oxidative phosphorylation, mitochondrial targeting, and fertilization-related processes (Fig. 3*M* and *SI Appendix*, Fig. S12*A*). These persistent signatures correspond to pathways critical for motility and energy metabolism and align with a modest but measurable increase in abnormal sperm morphology in recovery males compared with controls (Fig. 3*N* and *SI Appendix*, Fig. S12*B*). Thus, while meiotic and postmeiotic programs largely reestablish normal architecture and transcriptional coordination after withdrawal, residual transcriptional dysregulation persists in pathways essential for sperm function. These findings define a reversible yet functionally sensitive window of pachytene vulnerability that may underlie the transient contraceptive effect of JQ1.

### Full Fertility After Long-Term Drug Withdrawal and in F1 Pups From Fathers Treated With JQ1.

**Fig. 4.**
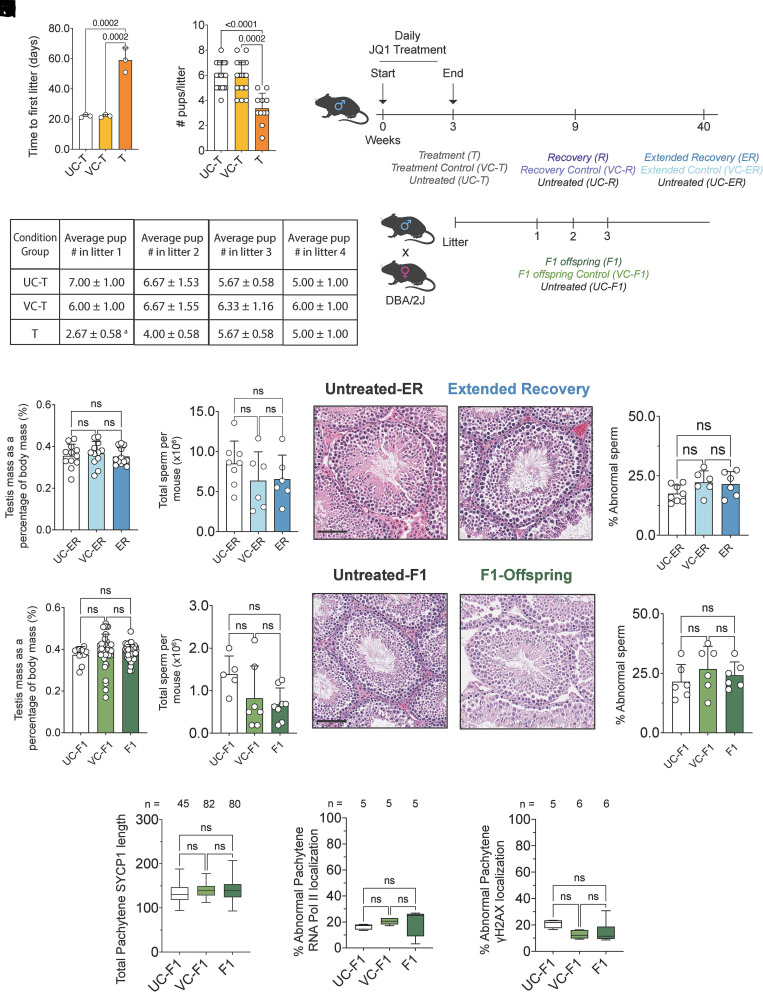
Fertility and offspring development fully recover following transient BRDT inhibition. (*A*) Time to first litter (days) for untreated (UC-T), vehicle (VC-T), and JQ1-treated (T) sires (n = 3 mice per group). (*B*) Average number of pups per litter from UC-T, VC-T, and T sires (n = 3 mice). Each point represents one litter; data are shown as mean ± SD. (*C*) Summary table showing mean litter size (±SD) across 4 consecutive litters from UC-T, VC-T, and T sires. Statistical comparisons were performed for each litter using the Kruskal–Wallis test with Dunn’s post hoc test; a, *P* = 0.0471 UC-T vs T. (*D*) Experimental design outlining breeding assay and condition groups for recovery (R), extended recovery (ER), and F1 analyses. Males received 3 wk of JQ1 or vehicle injections and were paired with DBA/2J females until producing three litters. Sires were analyzed at recovery (R, 6 wk), extended recovery (ER, 30 wk), or as F1 offspring (7 wk). (*E* and *F*) Testis mass expressed as a percentage of body mass (*E*) and total epididymal sperm counts (10^6^ sperm per mouse) (*F*) for extended-recovery males (UC-ER, n = 8; VC-ER, n = 8; ER, n = 8 mice). Data are shown as mean ± SD. Statistical comparisons were performed using one-way ANOVA; ns = not significant. (*G* and *H*) Representative H&E-stained testis sections from untreated (UC-ER) and extended-recovery (ER) males showing full spermatogenic restoration. (Scale bar, 100 µm.) (*I* and *J*) Testis mass (% of body mass) (*I*) and total epididymal sperm counts (10^6^ sperm per mouse) (*J*) for F1 males (UC-F1, n = 8; VC-F1, n = 8; F1, n = 8 mice). Data are shown as mean ± SD. Statistical comparisons were performed using the Kruskal–Wallis test with Dunn’s post hoc test; ns = not significant. (*K* and *L*) Representative H&E testis sections from untreated (UC-F1) and JQ1-derived (F1) males showing intact seminiferous architecture. (Scale bar, 100 µm.) (*M*) Quantification of abnormal sperm morphology (% ±SD) from UC-ER (n = 8), VC-ER (n = 8), and ER (n = 8) males. (*M* and *N*) Percentage of abnormal sperm per male from UC-ER (M) and UC-F1 (N) (n = 8 mice per group). Data are shown as mean ± SD. Statistical comparisons were performed using one-way ANOVA; ns = not significant. (*O*–*Q*) Meiotic integrity in F1 testes showing total pachytene SYCP1 length (n = 45 to 82 nuclei, 4 to 5 mice per group) (*O*), frequency of pachytene nuclei with autosomal RNA polymerase II (minimum 30 nuclei averaged across n = 5 mice) (*P*), and frequency with γH2AX signal outside the sex body (minimum 30 nuclei averaged across n = 5 to 6 mice) (*Q*). Boxplots show median and interquartile range. Statistical comparisons were performed using one-way ANOVA (*O* and *P*) or Kruskal–Wallis test with Dunn’s post hoc test (*Q*); ns = not significant.

Given the residual transcriptional alterations in pathways linked to sperm function, we next asked whether these changes translated into measurable effects on fertility. JQ1-treated, vehicle, and untreated males were paired with reproductively active wild-type DBA/2J females. Consistent with spermatogenic resumption following JQ1 withdrawal, treated males exhibited a short delay to first litter (Fig. 4*A*) and produced smaller initial litters with fewer pups per pairing compared with controls (Fig. 4 *B* and *C*). Subsequent breedings, however, yielded normal litter sizes and timing, indicating complete restoration of reproductive function once spermatogenesis normalized.

To evaluate long-term and transgenerational effects, we examined extended-recovery (ER; 30 wk post-JQ1 withdrawal) and first-generation (F1) offspring from the initial seven litters. Both ER and F1 males were compared with matched vehicle-treated and untreated controls. Testis mass, sperm counts, and testicular morphology were fully restored (Fig. 4 *E*–*L*), and sperm morphology was indistinguishable from controls (Fig. 4 *M* and *N*). Apoptotic indices also remained comparable across groups, with no increase in TUNEL-positive cells at either timepoint (*SI Appendix*, Fig. S5 *E* and *F*), confirming sustained testicular integrity. Moreover, chromosomal organization in F1 spermatocytes, including SC length and RNA polymerase II and γH2AX localization, was identical to that of controls (Fig. 4 *O*–*Q*).

Together, these data demonstrate that transient inhibition of BRDT during meiotic prophase I causes only a temporary reduction in fecundity, with complete recovery of fertility and normal offspring development. Functional restoration across both recovery and F1 generations provides strong evidence that meiosis can be pharmacologically and reversibly targeted without compromising long-term reproductive health, establishing the safety and tractability of this stage for contraceptive intervention.

### Transient BRDT Inhibition Modestly Alters CO Dynamics During Pachynema But Preserves Chiasmata Formation and Resolution.

A major safety consideration in targeting meiosis for contraception is the risk of aneuploidy, which arises when homologous chromosomes fail to segregate properly. Crossovers (COs), formed as the outcome of earlier prophase I recombination and synapsis events, are essential for establishing physical links between homologs. Mis-segregation often results from abnormal CO formation — either too few COs or their improper placement — since each synapsed chromosome pair must typically form at least one CO to ensure correct disjunction (7).

**Fig. 5.**
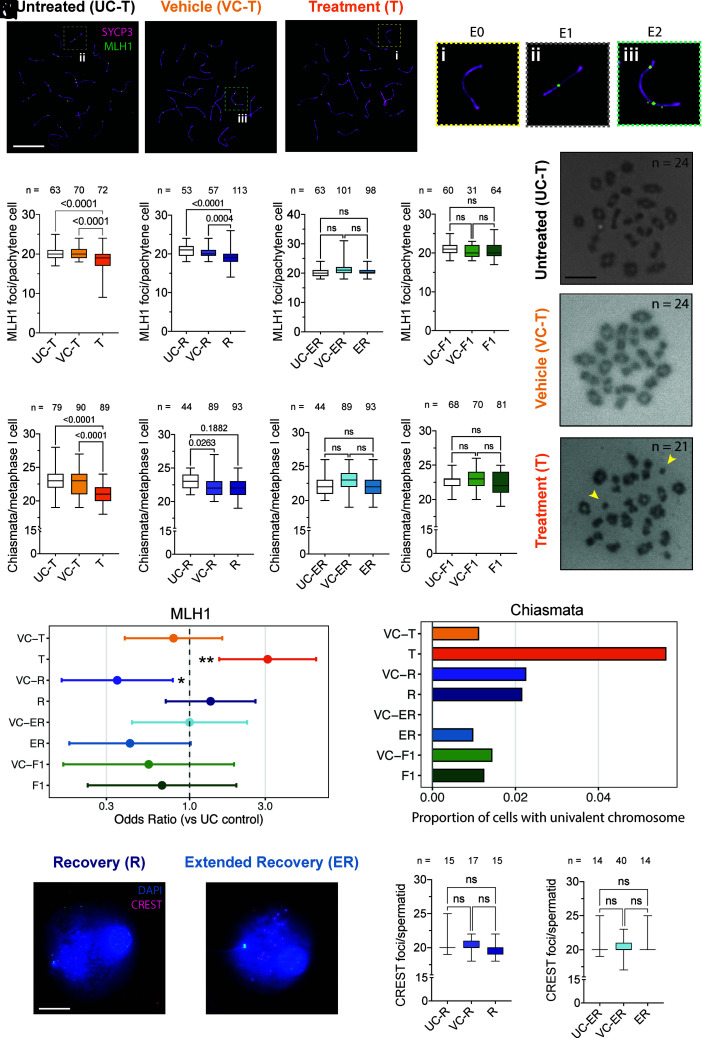
Transient BRDT inhibition reduces crossover number but preserves chromosomal integrity. (*A*–*C*) Representative pachytene spermatocytes from untreated (UC-T), vehicle-treated (VC-T), and JQ1-treated (T) males immunostained for SYCP3 (magenta) and MLH1 (green). Dashed boxes highlight individual SCs containing (Di) or lacking (Dii-iii) MLH1. (*D*) Individual SCs with zero (E0) (*i*), one (E1) (*ii*), or two (E2) (*iii*) MLH1 foci. (*E*–*H*) Quantification of MLH1 foci per pachytene nucleus in treatment (*E*) and recovery cohorts (*F*–*H*). Each point represents one nucleus; n = 31 to 113 nuclei pooled from 3 to 5 mice per group. Boxplots show median and interquartile range. Statistical comparisons were performed using the Kruskal–Wallis test with Dunn’s post hoc test; exact p-values are shown or ns = not significant. (*I*–*K*) Representative Giemsa-stained metaphase I spreads from UC-T (n = 24 crossovers), VC-T (n = 24 crossovers), and T (n = 21 crossovers) males. Yellow arrowheads indicate rare univalents. (Scale bars, 20 µm.) (*L*–*O*) Quantification of chiasmata per metaphase I cell across treatment (*L*) and recovery (*M*–*O*) cohorts. Each point represents one nucleus; n = 44 to 120 nuclei pooled from 3 to 5 mice per group. Boxplots show median and interquartile range. Statistical comparisons were performed using the Kruskal–Wallis test with Dunn’s post hoc test; exact p-values are shown or ns = not significant. (*P*) Generalized linear mixed-effects model (binomial) estimating odds ratios of MLH1-negative SCs relative to UC controls. Error bars denote 95% CI (*P < 0.05, **P < 0.01). (*Q*) Proportion of cells with univalent chromosomes from chiasmata spreads, analyzed by Fisher’s exact tests versus their matched controls (UC-T vs T p = 1.00; UC-R vs R p = 1.00; UC-ER vs ER p = 1.00; UC-F1 vs F1 p = 1.00; VC-T vs T p = 0.118; VC-R vs R p = 1.00; VC-ER vs ER p = 0.462; VC-F1 vs F1 p = 1.00). (*R* and *S*) Representative spermatid nuclei from recovery (R, *R*) and extended recovery (ER, *S*) cohorts immunostained for CREST (centromeres, magenta) and DAPI (blue). (Scale bar, 20 µm.) (*T* and *U*) Quantification of CREST foci per spermatid in R (*T*) and ER (*U*). Each point represents one spermatid; n = 14 to 40 cells pooled from 1 to 3 mice per group. Boxplots show median and interquartile range. Statistical comparisons were performed using the Kruskal–Wallis test with Dunn’s post hoc test; ns = not significant.

We quantified CO formation during pachynema using MLH1 foci, which mark nascent CO sites (19). Each homolog pair was categorized as E0, E1, or E2 based on the number of MLH1 foci present, corresponding to zero, one, or two CO sites per SC, respectively (Fig. 5 *D, i–iii*). Mature COs were evaluated at metaphase I by chiasmata counts (Fig. 5 *I*–*K*). MLH1 foci were modestly reduced in JQ1-treated males relative to untreated and vehicle controls (Fig. 5 *A*–*E* and *SI Appendix*, Fig. S13*A*), and odds-ratio analysis confirmed an increased frequency of chromosomes lacking an MLH1 focus (Fig. 5*P*). Despite this, among the limited cells that reached metaphase I, the proportion of fully bivalent cells was modestly reduced during treatment (*P* = 0.0438; *SI Appendix*, Fig. S13*E*), yet a generalized linear mixed model detected no increase in the odds of univalent chromosomes in T compared to UC-T (Fig. 5*Q*). These data indicate that homolog pairing and CO resolution remain sufficient to ensure faithful segregation even when total CO numbers are reduced, likely through the well-described secondary CO pathway (20).

Following drug withdrawal, MLH1 foci numbers partially recovered by 6 wk (Fig. 5*F*) and were indistinguishable from controls at the extended-recovery (ER; 30 wk) timepoint (Fig. 5 *G*–*H*). Chiasmata frequencies followed a similar trajectory, with full normalization by 6 wk and stable maintenance through ER and F1 generations (Fig. 5 *L*–*O*). Odds-ratio analysis further confirmed that the reduction in MLH1 foci observed during treatment was fully reversed after withdrawal (Fig. 5*P*). Consistent with these results, the proportion of pachytene nuclei with ≥1 MLH1-negative (E0) SC (*SI Appendix*, Fig. S13 *B*–*D*) and fully bivalent metaphase I cells (*SI Appendix*, Fig. S13 *F*–*H*) returned to control levels in R, ER, and F1 males. Across all recovery timepoints, the frequency of metaphase I cells containing univalents remained comparable to controls (Fig. 5*Q*), indicating no increase in aneuploidy resulting from JQ1 treatment.

To independently verify accurate chromosome segregation, we next quantified centromere number in haploid spermatids following recovery from JQ1 treatment using CREST immunostaining (21). Spermatids from short- and long-term recovery males displayed mostly the expected 20 centromere foci corresponding to the 20 mouse chromosomes, confirming normal segregation and the absence of aneuploidy at postmeiotic stages (Fig. 5 *R*–*T*), very much reducing (but not necessarily eliminating) concerns of aneuploidy in the ensuing spermatozoa population.

Together, these findings demonstrate that transient BRDT inhibition induces a mild alteration in CO processing during pachynema, but without compromising homolog alignment, CO maturation, or chromosome segregation. Crossover formation, bivalent structure, and meiotic fidelity are fully restored following JQ1 withdrawal and stably maintained across generations, underscoring the genomic safety of meiotic targeting as a contraceptive strategy.

## Discussion

The development of reversible, nonhormonal male contraceptives remains a critical unmet need for achieving reproductive equity. While multiple compounds have been shown to suppress sperm production, few studies define the molecular and cellular principles that distinguish a safe and reversible contraceptive target. Here, we use transient BRDT inhibition as a framework to test whether meiotic prophase I can be pharmacologically targeted and recovered without compromising long-term fertility or genomic integrity.

Our findings establish several criteria that characterize an effective meiotic contraceptive target. First, inhibition must produce a defined and stage-limited arrest without systemic toxicity or irreversible germ-cell loss. JQ1 satisfies this criterion as a test case: its activity is confined to pachynema, where transient attenuation of the transcriptional burst disrupts postmeiotic gene expression and predictably depletes spermatids and spermatozoa. Second, engagement must be transient and reversible. Accordingly, within one spermatogenic cycle after withdrawal, transcriptional programs, crossover frequency, and fertility fully recovered. Third, the drug intervention must preserve chromosomal integrity and preserve euploidy. Despite a temporary reduction in MLH1-marked crossover intermediates, we observe complete restoration of bivalent formation and haploid chromosome complements at the spermatid stage. With the caveat that we cannot wholly rule out the possibility of an increase in aneuploidy in the resulting spermatozoa, these results demonstrate that meiotic blockade can achieve the required transient, localized, reversible, and genetically stable requirements for safe contraceptive intervention.

This study extends current contraceptive frameworks, which predominantly act before or after meiosis. Early-stage strategies such as retinoic acid receptor inhibitors (e.g., YCT529) suppress spermatogonial differentiation (22), whereas posttesticular agents (e.g., sAC or SEMG1 inhibitors) impair sperm function (23, 24). These approaches, while effective, require continuous dosing and rarely engage the tightly regulated transitions of meiosis. By contrast, our data show that meiotic prophase I represents a precise and reversible control point—a stage where short-term perturbation halts gametogenesis without permanent loss of the underlying SSC pool.

Although our work focuses on the BET inhibitor JQ1 as a probe compound, its value lies in establishing BRDT and related chromatin readers as tractable models for reversible meiotic inhibition. The testis-specific expression of BRDT in both mouse (25) and human (26) makes it uniquely suited for pharmacologic targeting, but the pan-BET activity of JQ1 underscores the importance of selectivity in translational design. Because BRDT governs a broad transcriptional program required for meiotic and postmeiotic progression, its inhibition effectively targets entire gene networks rather than single effectors. Other BET paralogs (e.g., BRD4) may buffer transcriptional perturbations during treatment (27), explaining the mild and recoverable phenotype observed here compared with *Brdt* knockout models (9, 25, 28). Future efforts should refine compound specificity, enabling more specific control of meiotic transcription without off target BET inhibition. In addition, the need for comprehensive systemic safety assessment for any meiosis targeting drugs cannot be understated.

From a translational standpoint, these findings also highlight how duration and timing of inhibition determine both contraceptive efficacy and recovery. While fertility fully normalizes within 30 wk after short-term dosing, longer or continuous inhibition may yield cumulative effects not captured here. As male contraceptives would likely be used chronically, longitudinal studies across multiple spermatogenic cycles will be crucial to evaluate sustained reversibility and gamete quality.

Finally, this study provides functional molecular proof that meiosis can be targeted effectively for contraceptive development. Transient BRDT inhibition perturbs transcription, recombination, and spermatid maturation, yet all processes recover fully, producing chromosomally normal and fertile offspring. These findings establish a molecular roadmap for evaluating future meiotic targets—one grounded in measurable criteria for reversibility, genomic stability, and functional restoration. In sum, we demonstrate that meiotic prophase I is a pharmacologically accessible and reversible stage in spermatogenesis. By coupling temporal molecular precision with comprehensive recovery genomic profiling, this work provides a framework for developing meiosis-based male contraceptives that are both effective and inherently reversible, advancing the field toward practical, equitable solutions for fertility control.

## Materials and Methods

### Mice.

All experiments were performed using DBA/2J mice (*Mus musculus*, Jackson Laboratory, strain #000671). Male mice were 7 wk old at the start of experiments. Female mice used for breeding were 8 wk old. Additional age-matched control males (7, 10, 15, and 34 wk) were obtained from Jackson Laboratory. Mice were housed under controlled temperature and light, with food and water provided ad libitum. All animal procedures were approved by the Cornell Institutional Animal Care and Use Committee (protocol #2004-0063).

### JQ1 Dosage and Injections.

We used a previously described treatment regimen (10) in which JQ1 (AdooQ Bioscience, CAS #1268524-70-4) was dissolved in DMSO at 50 mg/mL and diluted 1:10 in 10% (2-Hydroxypropyl)-β-cyclodextrin (Sigma-Aldrich, H5784). JQ1 was administered by intraperitoneal (i.p.) injection at 1% of body weight daily for 3 wk. Vehicle control mice received the same regimen using DMSO diluted 1:10 in 10% (2-Hydroxypropyl)-β-cyclodextrin. All injected mice were weighed daily prior to dosing, and injection volumes were adjusted accordingly. Body weight trajectories are shown in *SI Appendix*, Fig. S1. Injected volumes for each mouse are provided in Dataset S1.

### Breeding Assay.

To assess fertility, male mice (n = 3 per group) were paired for breeding immediately following 3 wk of JQ1 or vehicle treatment. Age-matched untreated 10-week-old DBA/2J males from Jackson Laboratory served as additional controls. Each treated or control male was housed continuously with a DBA/2J female for 14 d, without monitoring for copulatory plugs. A single nulliparous female was introduced every 14 d for a total of seven breeding cycles (14 wk). Sires received no additional treatment during the breeding period. Male offspring from each litter were weaned upon sexual maturity and maintained under identical housing conditions until analysis at 7 wk of age. All raw breeding data, including litter counts, timing, and number of pups, are provided in *SI Appendix*, Supplementary Data, Sheet 23.

### Single Cell Testicular Suspension.

One testis per animal (n = 3 per group) was collected for single-cell RNA sequencing. Testes were detunicated, minced on ice, and transferred to 40 mL of digestion buffer containing collagenase IV (1 mg/mL, Sigma-Aldrich, C4-BIOC) and DNase I (7 mg/mL, Sigma-Aldrich, DN25) in DMEM (Gibco, 11-965-092). Samples were incubated at 37 °C for 15 min, centrifuged at 60×*g* for 10 min, and the supernatant discarded. Tubules were washed once with 15 mL Dulbecco’s phosphate-buffered saline (dPBS; Sigma-Aldrich, D8537) and centrifuged at 60×*g* for 10 min. The pellet was resuspended in 10 mL 0.05% trypsin-EDTA (Invitrogen, 25200056) and incubated at 37 °C for 12 min. Trypsin activity was quenched with 10% (v/v) fetal bovine serum (FBS; Thermo Fisher Scientific, A5256701). Cells were passed through a 40 µm filter, centrifuged at 300×*g* for 5 min, and resuspended in 1 mL of 0.04% bovine serum albumin (BSA; Sigma-Aldrich, A4737) in 1× dPBS. Single-cell suspensions were submitted to the Cornell DNA Sequencing Core Facility for 10x Genomics Chromium library preparation and sequencing.

### Single Cell RNA Sequencing.

#### Library preparations and sequencing.

Single-cell RNA-seq libraries were prepared using the Chromium Single Cell 3′ v3 platform (10× Genomics). At each time point, approximately 8,000 cells were captured per library (n = 3 mice per condition). In total, 15 libraries were generated, pooled at equimolar concentrations, and sequenced across one lane of an Illumina NovaSeq 6000 (Novogene, CA) to minimize batch effects. Sequencing depth and saturation metrics are summarized in *SI Appendix*, Table S1.

#### Preprocessing and quality control of scRNA-seq datasets.

Raw sequencing data were processed using the Cell Ranger count pipeline (v3.0.0) against the *Mus musculus mm10* reference genome using STAR (v2.5.3b) (29).

Downstream analyses were performed in R using Seurat (v4.1.1) (30). Ambient RNA contamination was corrected using SoupX (v1.4.5) (31), and putative doublets were identified and removed with DoubletFinder (v2.0) (32) following 10x Genomics recommendations. Standard quality control filters were applied to exclude low-quality cells based on gene detection, total transcript count, and mitochondrial transcript proportion (*SI Appendix*, Fig. S2*B*). After filtering, samples were merged into a single Seurat object for downstream analyses. Because all samples were processed and sequenced concurrently, batch correction was not required prior to merging.

#### Outlier identification and handling.

One scRNA-seq treatment-control replicate (TC1) was identified as an outlier based on PCA (Fig. 3*G*) and reduced pairwise Pearson correlation with other control samples (*SI Appendix*, Fig. S2*D*; *r* = 0.93 to 0.94). Examination of per-sample QC metrics confirmed that this deviation reflected technical rather than biological variation (*SI Appendix*, Fig. S2*E*). To prevent inflation of between-group correlations, TC1 was excluded from differential-expression analyses but retained for unsupervised approaches (e.g., clustering, pseudotime inference, and PCA visualization). This approach also enabled consistent differential testing of Treatment (T) and Recovery (R) groups against a shared untreated control (UC).

#### Cell type annotation.

Cell type identities were assigned based on canonical marker gene expression (*SI Appendix*, Figs. S3 and S4). Clusters displaying highly similar expression patterns were merged to improve biological interpretability. Clustering at a resolution of 1.2 initially produced 32 clusters; after merging related groups, eight major cell types and 21 subclusters were defined, encompassing all stages of spermatogenesis. Cluster annotations were confirmed by correlation with reference testis scRNA-seq datasets (5, 6).

#### Pseudotime workflow.

To infer germ cell developmental trajectories, the merged Seurat object was subset to include only germ cells. Pseudotime ordering was performed using Slingshot (v2.4.0) (12) with UMAP coordinates as the reduced-dimensional input and cluster labels as lineage identifiers. Pseudotime values were extracted using slingPseudotime, and trajectories were visualized using the SlingshotDataSet function. Cells were binned into 51 pseudotime intervals to facilitate expression trend analyses across spermatogenesis (*SI Appendix*, Fig. S7).

#### DE analysis.

Bulk-like DE testing was performed using the AggregateExpression function in Seurat to compute sample-level averages by condition. DE between conditions was assessed using DESeq2 (33) through Seurat’s FindMarkers interface. Genes were considered differentially expressed if they met all of the following criteria: adjusted p < 0.05 (FDR) and average |log_2_ fold change| > 1. Raw DE results for all germ-cell subsets are provided in Dataset S8 and *SI Appendix*, Tables 1–8.

A “healing” metric was computed to quantify transcriptional recovery, defined as the proportion of genes differentially expressed during treatment (T vs UC) that were no longer significantly altered after withdrawal (R vs UC). This reflects the fraction of treatment-induced transcriptional changes that returned to baseline. Raw values are provided in Dataset S16.

### Testis Mass Quantification.

For each animal, each testis was weighed independently and combined to calculate the proportion of testis mass:body mass. Raw testis and body mass measurements are provided in Dataset S2.

### Sperm Capture Methods.

#### Total epididymal sperm collection.

Caudal epididymides from untreated, vehicle-treated, or JQ1-treated males were dissected into Toyoda–Yokoyama–Hoshi (TYH) medium. Samples were incubated at 37 °C for 20 min to allow sperm to swim out into the medium. Aliquots were diluted 1:25 in 10% neutral buffered formalin, and spermatozoa were counted using a standard hemocytometer. Morphological abnormalities were assessed by light microscopy. Raw sperm count data are provided in Dataset S3, and morphology data are provided in Dataset S6.

#### Hematoxylin-eosin staining of epididymal sperm smears.

Ten microliters of total epididymal sperm were mixed with 10 µL of 4% paraformaldehyde (PFA), spread on glass slides, and air-dried for 6 h at room temperature. Slides were stored at 4 °C until use, then washed in 1× PBS and stained with hematoxylin and eosin following standard protocols.

### Prophase I Chromosome Spreading, Immunofluorescence, and Quantitation.

Chromosome spreads were prepared as previously described (20). Primary antibodies were diluted in antibody dilution buffer (ADB; 3% BSA, 1% goat serum [Gibco, 16210-072], 0.05% Triton X-100 in PBS) and incubated overnight at room temperature. The following primary antibodies were used: rabbit anti-SYCP3 [1:1,000; custom-made (34)], mouse anti-γH2AX (1:10,000; EMD Millipore, 05-636), mouse anti-SYCP3 (1:1,000; Abcam, ab97627), mouse anti-MLH1 (1:50; BD Pharmingen, 550838), rabbit anti-RAD51 (1:500; Millipore, PC130), mouse anti-RNA polymerase II (1:2,000; Sigma-Aldrich, 05-623), and rabbit anti-BRDT (1:500; Abcam, ab286194). After primary incubation and washing in 10% ADB, slides were then incubated for 2 h at room temperature with Alexa Fluor-conjugated secondary antibodies (Jackson ImmunoResearch, 1:1,000 in ADB): goat anti-mouse IgG Fc (AF488, 115-546-008; AF594, 115-586-008) and goat anti-rabbit IgG Fc (AF488, 111-546-046; AF594, 111-586-046). Slides were washed, air-dried, and mounted with ProLong Gold Antifade containing DAPI (Invitrogen, P36962).

### Quantification of Prophase Markers.

Chromosome spreads were immunostained for γH2AX and SYCP3 to stage spermatocytes across prophase I. The first 100 nuclei per animal were scored to determine the proportion of cells at each substage. RAD51 foci were quantified in zygotene spermatocytes, and only foci colocalized along SYCP3-positive SCs were recorded. γH2AX distribution was assessed in pachynema, with cells classified as abnormal if γH2AX signal extended beyond the sex body into autosomes. RNA polymerase II exclusion was evaluated by DAPI costaining; cells were considered abnormal if Pol II signal was not excluded from the sex body. SC length was measured using a FIJI macro that identified SYCP1-labeled SCs, and the summed autosomal SC length was reported per cell. MLH1 foci colocalizing with SYCP3 were counted and reported as total foci per cell, and the percentage of cells lacking MLH1 foci was also calculated. The FIJI macro used for SC length quantification is provided in the *SI Appendix*, *Methods*. All raw scoring data across antibodies are available in Datasets S10–S14 and S16.

### Diakinesis Preparations to Observe Chiasmata.

Diakinesis chromosome spreads were prepared as previously described (35), stained for 6 min in 4% Giemsa (Fisher), rinsed three times with double-distilled H_2_O for 3 min each, air-dried, and mounted with Permount (Fisher Scientific). Raw chiasmata count data for all experimental groups are provided in Dataset S17.

### CREST Staining and Counting.

To determine round spermatid aneuploidy rates, chromosome spread preparations were costained with primary antibodies against SYCP3 (1:1,000 dilution; Abcam, Cat. Number ab97672), ACRV1 (1:500 dilution; Proteintech, Cat. Number 14040-1-AP), and CREST (1:1,000 dilution; Antibodies incorporated, Cat. Number 15-234) and counterstained with DAPI. CREST-positive foci were analyzed only in ACRV1-positive round spermatids as outlined in Oppedisano *et al.* (21) Briefly, centromere “foci” were classified as singletons or clumps based on diameter and intensity. The size of each singleton centromere was used to estimate the number of centromeres per clump (typically up to 4), and counts were confirmed by similar quantitation of DAPI-bright foci. Foci positive for both ACRV1 and CREST were considered background acrosome staining, as previously described (21) and excluded from total counts. Given the variability in nonspecific CREST staining, DAPI quantification provided confidence of accuracy. Cells with fewer than 15 CREST-positive foci were excluded (21).

### Histological Methods.

One testis from each mouse was collected across all experimental groups and fixed in 10% neutral-buffered formalin overnight at room temperature, washed extensively in graded ethanol, paraffin-embedded, sectioned at 5 µm and processed for hematoxylin and eosin staining or immunohistochemistry.

Immunofluorescence was performed as described previously (20). Slides were incubated with primary antibodies for 1 h at 37 °C. The following primary antibodies were used: mouse anti-SYCP3 (1:1,000), rabbit anti-BRDT (1:500), and Lectin PNA-Alexa Fluor 647 conjugate (1:500; Invitrogen, L32460). After washing in PBS, slides were incubated with fluorescence-conjugated secondary antibodies (1:500) for 1 h at 37 °C, washed again, and mounted using ProLong Gold Antifade with DAPI (Invitrogen).

Seminiferous tubules were categorized into stages I, II–III, IV–VI, VII–VIII, IX–X, and XI–XII based on lectin staining patterns and the composition of germ cell types. Raw staging data for all samples are provided in Dataset S4. Raw Sertoli counts per seminiferous tubule is provided in Dataset S7.

### Image Acquisition.

Immunofluorescence images were acquired on a Zeiss Axio Imager epifluorescence microscope using a 63X oil-immersion objective. Images were processed in Zeiss Zen Blue v3.0 (Carl Zeiss AG, Oberkochen, Germany), and background levels were standardized using ImageJ (NIH). Exposure settings were kept constant across all samples and experimental conditions. Diakinesis preparations stained with Giemsa were imaged at 40X magnification in brightfield mode.

### Statistical Analysis.

Statistical analyses were conducted using GraphPad Prism version 10.0 (GraphPad Software, San Diego, California, USA). Data normality was assessed using the Shapiro–Wilk test. For normally distributed data, one-way ANOVA was applied; when normality assumptions were not met, the nonparametric Kruskal–Wallis test was used, followed by Dunn’s post hoc multiple-comparison test where appropriate. Fisher’s exact test was used for categorical comparisons (e.g., proportions of abnormal cells, presence of univalents). Generalized linear mixed models were used to assess treatment effects while accounting for replicate variability. Statistical significance was defined as α = 0.05, and all tests were two-sided. Raw input for generalized linear mixed models is provided in Dataset S19.

## Supplementary Material

Appendix 01 (PDF)

Dataset S01 (XLSX)

Dataset S02 (XLSX)

Dataset S03 (XLSX)

Dataset S04 (XLSX)

Dataset S05 (XLSX)

Dataset S06 (XLSX)

Dataset S07 (XLSX)

Dataset S08 (XLSX)

Dataset S09 (XLSX)

Dataset S10 (XLSX)

Dataset S11 (XLSX)

Dataset S12 (XLSX)

Dataset S13 (XLSX)

Dataset S14 (XLSX)

Dataset S15 (XLSX)

Dataset S16 (XLSX)

Dataset S17 (XLSX)

Dataset S18 (XLSX)

Dataset S19 (XLSX)

## Data Availability

All sequencing data generated in this study have been deposited in the Gene Expression Omnibus (GEO) repository through GEO series accession numbers GSE310340 (36) and GSE310342 (37). All codes have been deposited to GitHub (38).
