# Supplementary material for: Meiotic prophase I disruption as a strategy for nonhormonal male contraception using small-molecule inhibitor JQ1
Source: Proc Natl Acad Sci U S A. 2026 Apr 7;123(15):e2517498123. doi: 10.1073/pnas.2517498123 (PMC13080027; doi:10.1073/pnas.2517498123)
Supplement: Supplementary file 1 — Appendix 01 (PDF) [file pnas.2517498123.sapp.pdf]

SI Appendix

RNA-seq Methods

RNA-seq library preparation and sequencing. Total RNA was extracted from frozen liver and kidney samples using TRIzol Reagent (Thermo Fisher Scientific, 15596018) following the TREx RNA Extraction Protocol v1.8 (Cornell University Transcription Regulation and Expression Facility). After tissue homogenization, chloroform was added to promote phase separation of RNA from DNA and protein, and the aqueous phase was transferred to Phaselock Gel tubes (VWR 01847-802) for clean extraction. The RNA was precipitated with isopropanol (molecular biology grade), using GlycoBlue (Thermo AM9515) to enhance recovery. Pellets were washed twice with 75% ethanol, air-dried, and resuspended in RNase-free water. RNA concentration was quantified using a NanoPhotometer N50 (Implen) and RNA integrity assessed with an Agilent 2100 Bioanalyzer. Libraries were generated using the NEBNext UltraExpress RNA Library Prep Kit (NEB #E3330), incorporating poly(A) enrichment to select mRNA. Sequencing was performed across one lane of an Illumina NextSeq 2000 using single-end 75 bp reads, targeting ~20-25 million reads per sample. Each experimental group included 3 Treatment samples, 3 Recovery samples, and 4 Control samples per organ (3 Control from the treatment timepoint, 1 from the recovery timepoint).

Data QC and transcript quantification. Raw sequencing reads (FASTQ) were trimmed to remove adaptor sequences and low-quality bases using fastp (v0.20.1) (1) with default settings. Transcript-level quantification was performed using Salmon (v1.10.0) in quasi-mapping mode against the mm10 transcriptome (GENCODE vM30). Gene-level counts were generated using tximport (v1.26.1) (2) with the option countsFromAbundance="lengthScaledTPM" to account for transcript length and sequencing depth.

Differential gene expression analysis. Count data were modeled using DESeq2 (v1.38.3) (3) with the design formula ~condition, where condition was defined as Treatment, Recovery, or Control, analyzed separately for liver and kidney. Dispersion estimates and size factors were calculated following the standard workflow. Differentially expressed genes were identified using the Wald test, and multiple testing correction performed using the Benjamini-Hochberg procedure. Genes with an adjusted p-value < 0.05 were considered differentially expressed.

Sample similarity was assessed by calculating pairwise Pearson correlations between rlog-transformed counts across all samples, which were visualized as a heatmap with annotations for organ and experimental condition to confirm clustering of biological replicates.

Code and data availability. All raw and processed bulk RNA-seq data generated for this study has been deposited in the Gene Expression Omnibus (GEO) as GSE310340. Analysis scripts used to process data and generate figures are available through a GitHub repository (https://github.com/CohenLab-Cornell/JQ1_testis_RNAseq).

TUNEL staining of testicular sections.

TUNEL staining was performed using a TUNEL staining kit (Apoptag kit, EMD Millipore, S7100) following the manufacturer's instructions. Images were obtained using ImageScope (Leica Biosystems), and DAB-positive cells were manually counted using Adobe Photoshop software. The number of apoptotic cells was quantified as the total number of DAB-positive cells divided by the total number of tubules per testis section. One section per animal was analyzed, and scorers were not blinded to experimental condition. Raw TUNEL scores are provided in Supplementary Data, Sheet 5.

FIJI ImageJ macro script for measuring chromosome length with SYCP1

An Image J macro script was created to measure the length of the synaptonemal complex using the available tools in ImageJ. Images were in .czi file format, with SYCP3 in green and SYCP1 in red. The length of the central element of the synaptonemal complex, inferred from SYCP1, was measured in pachytene staged cells. The script used was as follows:

```
//input:chosen directory in which czi files are located

//output: data.txt in the same directory with fname,roi#,length (tab delineated),

//and roi.zips for each image

//User adjustable paramaters

alpha=0.8; //Number of standard deviations above the median for the threshold

cw=0.9; //Average chromosome width for translating perimeter to length
```

```
mcs=2.0; //Min chromosome area (in image units) for filtering out noise

dir=getDir("Choose Directory in which data files are stored");
print(dir);
files=getFileList(dir);
fp=File.open(dir+"data.txt");
print(fp, "fname\troi#\tlength");
for (k = 0; k < files.length; k++) {
	iCzi=indexOf(files[k],".czi");
	if (iCzi != -1) {
		fname=substring(files[k],0,iCzi);
		run("Bio-Formats", "open="+dir+files[k]+" color_mode=Composite");
		run("Split Channels");
		close("\\Others");
		run("Unsharp Mask...", "radius=20 mask=0.9");
		med=getValue("Median");
		stdev=getValue("StdDev");
		thresh=med+alpha*stdev;
		maxBD=bitDepth();
		maxBD=Math.pow(2,maxBD)-1;
		setThreshold(thresh, maxBD);
```

```
        run("Convert to Mask");
        run("Analyze Particles...", "size="+mcs+"-Infinity exclude clear add");
        roiManager("Save", dir+fname+".zip");
        nroi=roiManager("count");
        for (i = 0; i < nroi; i++) {
            roiManager("select", i);
            perim=getValue("Perim.");
            len=(perim-PI*0.9)/2.0;
            print(fp, fname+"\t"+i+1+"\t"+len);
            roiManager("deselect");
            }
        close();
        }
}
File.close(fp);
run("Close");
```

Supplementary Figures

Tanis, Simon, et al., Supplemental Figure 1

A

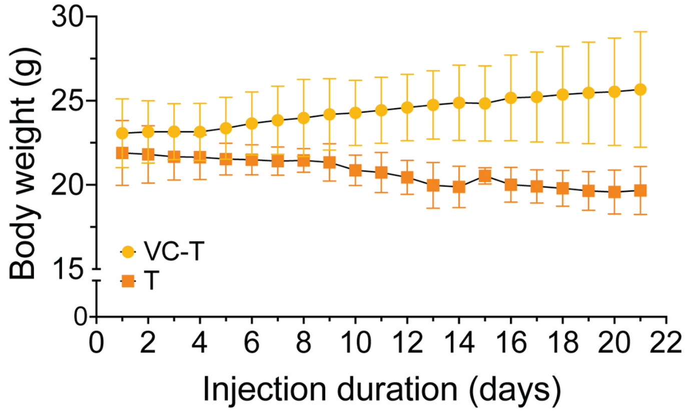

Figure S1. Body-weight monitoring during daily JQ1 treatment.

(A) Mean ± SD body weight of vehicle-treated (VC-T) and JQ1-treated (T) males measured prior to each daily intraperitoneal injection over 21 consecutive days. JQ1 (50 mg/mL in DMSO, diluted 1:10 in 10% (2-Hydroxypropyl)-β-cyclodextrin) or vehicle was administered at 1% of body weight per day, with dosing volumes adjusted to same-day weight. Body-weight trajectories remained stable throughout the treatment period.

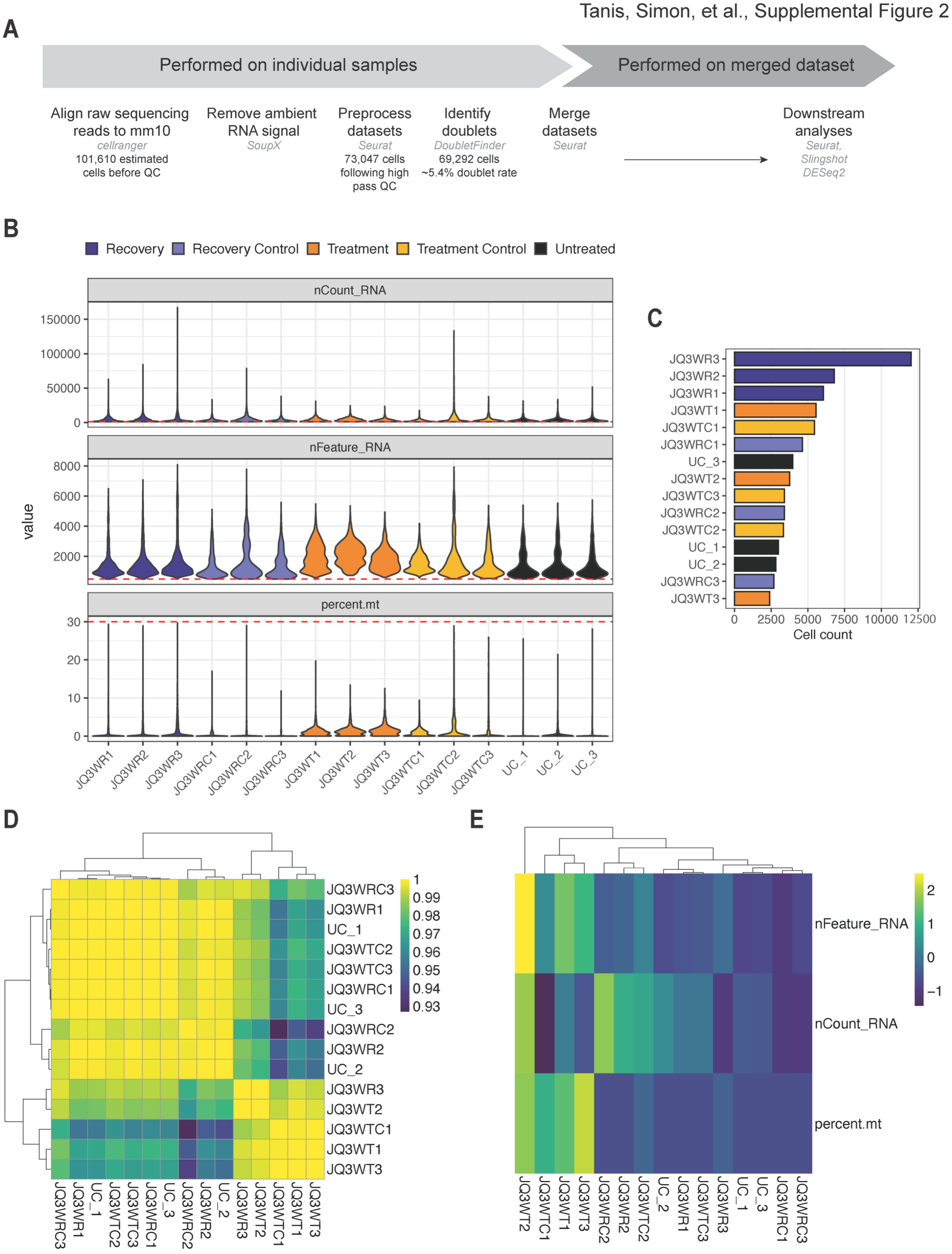
A
Performed on individual samples
Performed on merged dataset
Align raw sequencing reads to mm10
cellranger
101,610 estimated cells before QC
Remove ambient RNA signal
SoupX
Preprocess datasets
Seurat
73,047 cells following high pass QC
Identify doublets
DoubletFinder
69,292 cells
~5.4% doublet rate
Merge datasets
Seurat
Downstream analyses
Seurat, Slingshot DESeq2
B
Recovery
Recovery Control
Treatment
Treatment Control
Untreated
nCount_RNA
nFeature_RNA
percent.mt
value
JQ3WR1
JQ3WR2
JQ3WR3
JQ3WRC1
JQ3WRC2
JQ3WRC3
JQ3WT1
JQ3WT2
JQ3WT3
JQ3WTC1
JQ3WTC2
JQ3WTC3
UC_1
UC_2
UC_3
C
Cell count
D
E
nFeature_RNA
nCount_RNA
percent.mt

Figure S2. Preprocessing, quality control, and outlier identification of single-cell RNA-seq libraries. (A) Overview of the preprocessing workflow. Raw sequencing reads were aligned to the Mus musculus (mm10) reference genome using Cell Ranger (v3.0.0). Ambient RNA contamination was removed with SoupX (v1.4.5), and each dataset was independently filtered and normalized in Seurat (v4.1.1). Putative doublets were identified with DoubletFinder (v2.0), and high-confidence cells were merged into a single Seurat object for downstream analyses. (B) Violin plots showing distributions of detected features (nFeature_RNA), total transcript counts (nCount_RNA), and mitochondrial read percentages (percent.mt) for each library following quality filtering. Red dashed lines indicate the applied QC thresholds. (C) Total number of high-quality cells retained per library after preprocessing and doublet removal. (D) Pearson correlation heatmap of median QC metrics across libraries, demonstrating overall consistency among replicates. (E) Z-score heatmap of nFeature_RNA, nCount_RNA, and percent.mt across libraries, showing deviation from the cohort mean. One treatment-control library (JQ3WTC1; TC1) exhibits low total counts (nCount_RNA, dark blue), average to slightly below-average detected features (nFeature_RNA), and no notable shift in mitochondrial percentage, a discordant pattern relative to other replicates. This indicates a likely technical artifact (e.g., reduced library complexity or depth). TC1 was excluded from differential-expression analyses but retained for unsupervised visualization/clustering.

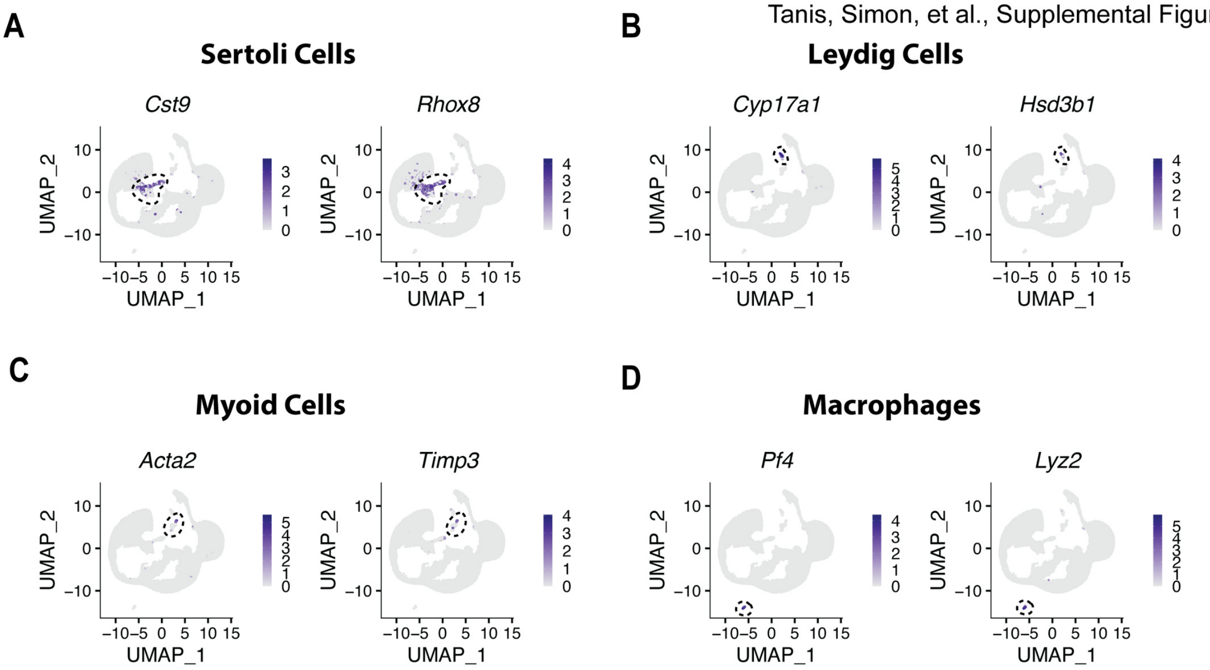

Figure S3. Somatic cell marker expression defines supporting and interstitial populations.

(A–D) UMAP visualization of somatic cell clusters from the integrated testis single-cell dataset, showing expression of canonical markers for each cell type. (A) Sertoli cells (*Cst9, Rhox8*), (B) Leydig cells (*Cyp17a1, Hsd3b1*), (C) Myoid cells (*Acta2, Timp3*), and (D) Macrophages (*Pf4, Lyz2*). Gene expression is shown as normalized expression (color scale) overlaid on the UMAP. Dashed outlines indicate the approximate location of each somatic cell population.

Tanis, Simon, et al., Supplemental Figure 4

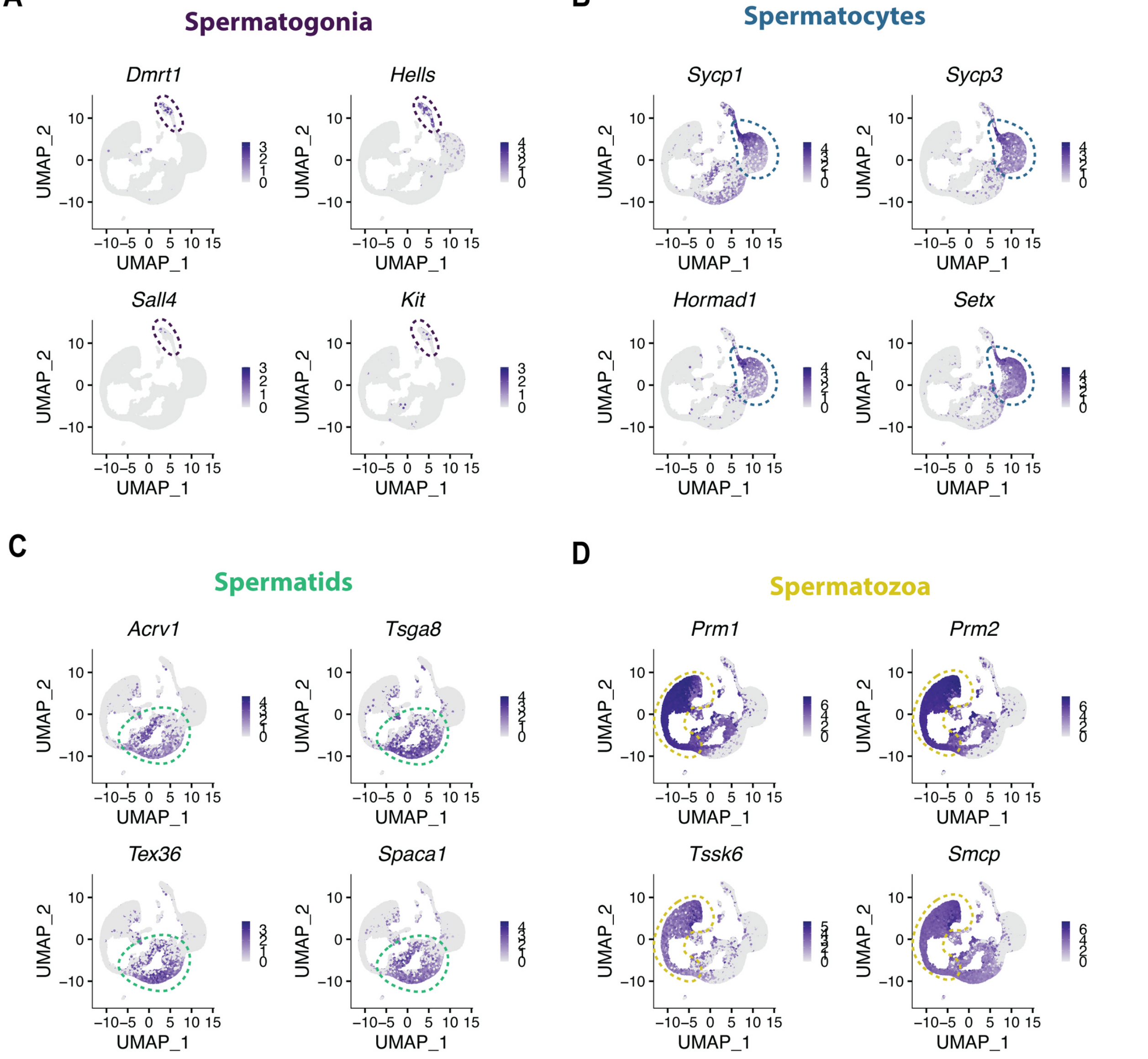

Figure S4. Stage-specific marker gene expression defines germ cell subtypes across spermatogenesis. (A–D) UMAP visualization of germ cell clusters from the integrated testis single-cell dataset, showing expression of representative marker genes for each developmental stage. (A) Spermatogonia markers (*Dmrt1, Hells, Sall4, Kit*), (B) Spermatocytes markers (*Sycp1, Sycp3, Hormad1, Setx*), (C) Spermatids markers (*Acrv1, Tsga8, Tex36, Spaca1*), and (D) Spermatozoa markers (*Prm1, Prm2, Tssk6, Smcp*). Gene expression is shown as normalized expression (color scale) overlaid on the UMAP. Dashed outlines correspond to the approximate spatial domains of each germ cell stage, colored according

to the respective label titles (*Spermatogonia*, purple; *Spermatocytes*, blue; *Spermatids*, green; *Spermatozoa*, gold).

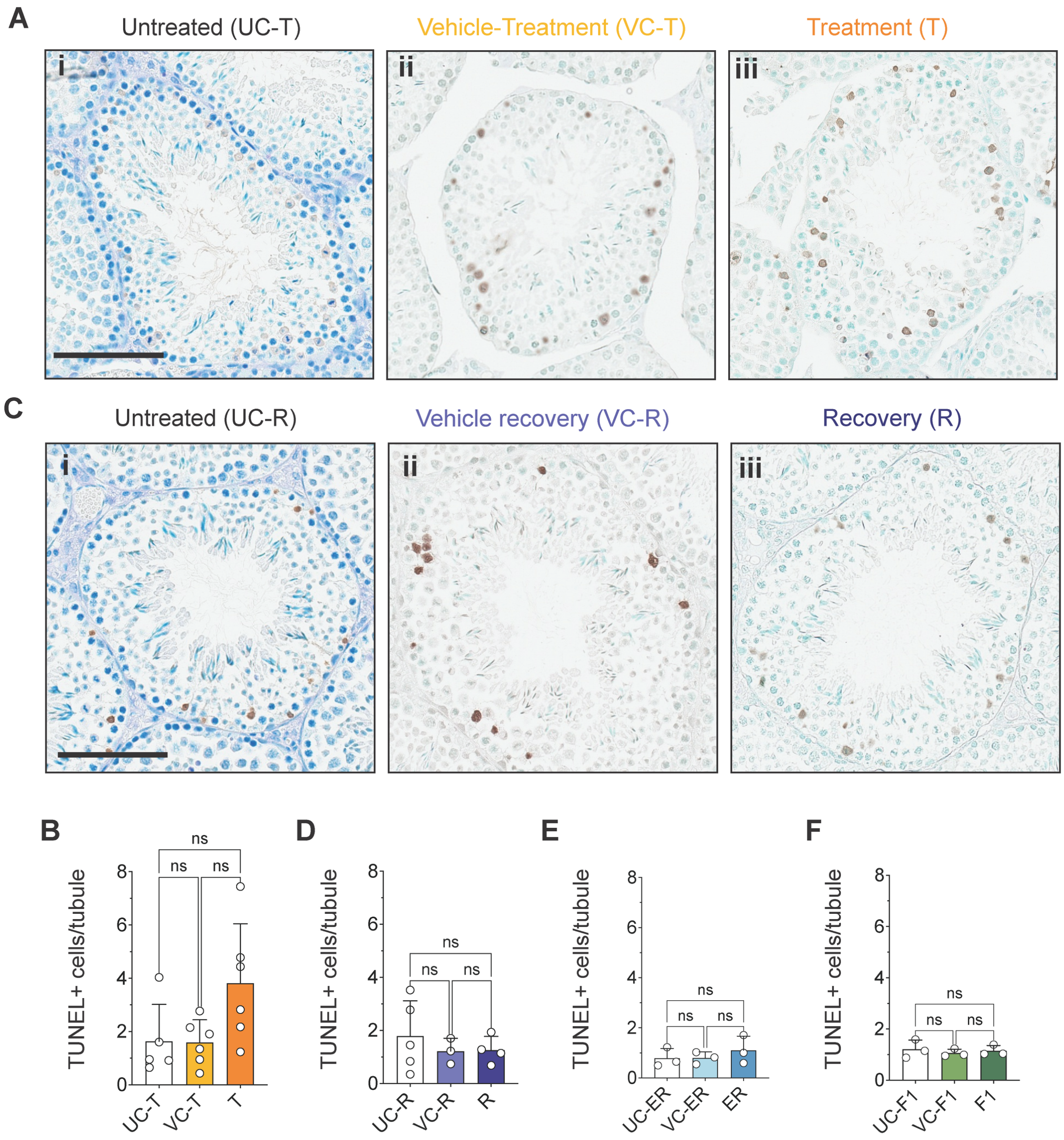

Figure S5. Limited apoptotic response following short-term JQ1 treatment and full recovery after withdrawal. (A–B) Terminal deoxynucleotidyl transferase dUTP nick-end labeling (TUNEL) assay showing DAB-positive apoptotic cells (brown) counterstained with Methyl Green in seminiferous tubules from (i) untreated (UC-T), (ii) vehicle-treated (VC-T), and (iii) JQ1-treated (T) males. Quantification of TUNEL-

positive cells per tubule is shown in (B) for each condition (UC-T, n = 5; VC-T, n = 6; T, n = 6). (C–D) Equivalent analyses following recovery, showing representative images from (i) untreated (UC-R), (ii) vehicle recovery (VC-R), and (iii) recovery (R) males, and corresponding quantification (D; UC-R, n = 5; VC-R, n = 3; R, n = 4). (E–F) Analyses extended to the extended recovery (ER) and first-generation (F1) timepoints (UC-ER, n = 3; VC-ER, n = 3; ER, n = 3; UC-F1, n = 3; VC-F1, n = 3; F1, n = 3). R and ER groups passed normality tests and were analyzed by one-way ANOVA; T and F1 groups did not and were analyzed using the Kruskal–Wallis test (ns = not significant). Scale bars, 100 μm.

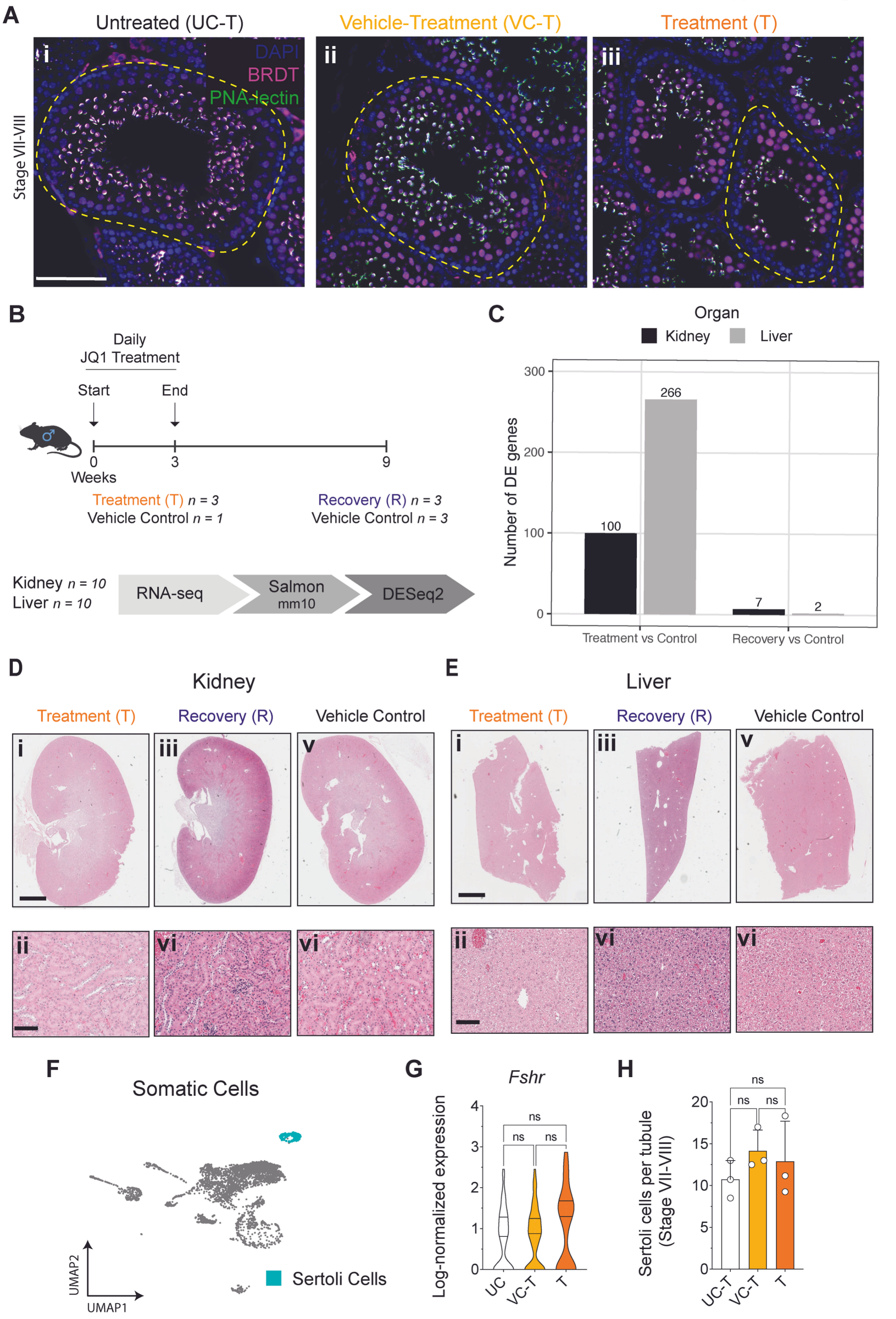
A
Untreated (UC-T)
Vehicle-Treatment (VC-T)
Treatment (T)
i
ii
iii
DAPI
BRDT
PNA-lectin
Stage VII-VIII
B
Daily
JQ1 Treatment
Start
End
0
3
9
Weeks
Treatment (T) n = 3
Vehicle Control n = 1
Recovery (R) n = 3
Vehicle Control n = 3
Kidney n = 10
Liver n = 10
RNA-seq
Salmon
mm10
DESeq2
C
Organ
Kidney
Liver
Number of DE genes
300
200
100
0
266
100
7
2
Treatment vs Control
Recovery vs Control
D
Kidney
Treatment (T)
Recovery (R)
Vehicle Control
i
iii
v
ii
vi
vi
E
Liver
Treatment (T)
Recovery (R)
Vehicle Control
i
iii
v
ii
vi
vi
F
Somatic Cells
Sertoli Cells
UMAP2
UMAP1
G
Fshr
Log-normalized expression
4
3
2
1
0
ns
ns
ns
UC
VC-T
T
H
Sertoli cells per tubule
(Stage VII-VIII)
20
15
10
5
0
ns
ns
ns
UC-T
VC-T
T

Figure S6. JQ1 acts as a testis-specific BRDT inhibitor with minimal off-target or hormonal effects. (A) Immunofluorescence staining for BRDT (green) and PNA-lectin (acrosome, magenta) in stage VIII–XII seminiferous tubules from untreated (UC-T), vehicle-treated (VC-T), and JQ1-treated (T) males. Yellow dashed lines outline a representative seminiferous tubule. BRDT protein remains detectable in spermatocytes and spermatids following treatment, consistent with JQ1 acting as a competitive rather than degradative inhibitor. (B) Experimental design of the RNA-seq cohort showing daily JQ1 or vehicle treatment for three weeks followed by a three-week recovery period. Samples collected at the treatment (T) and recovery (R) timepoints were compared to a combined control group (one vehicle control at T and three vehicle controls at R). The general RNA-seq analysis workflow is shown below (see Supplementary Methods for details). (C) Differential expression analysis of kidney and liver RNA-seq data. While JQ1 treatment induced a modest number of differentially expressed genes in the kidney and very few in the liver, both organs showed near-complete recovery to baseline expression following treatment withdrawal. (D–E) Representative H&E staining of kidney (D) and liver (E) from treatment, recovery, and vehicle control males showing preserved tissue architecture at both low magnification (i, iii, v) and higher magnification (ii, iv, vi). (F) UMAP of somatic cells from the integrated testis scRNA-seq dataset, highlighting Sertoli cells (cyan) identified using marker genes from Supplementary Figure 3A. (G) Violin plots of *Fshr* expression in Sertoli cells from UC-T (77 cells), VC-T (112 cells), and T (24 cells) scRNA-seq libraries, showing no significant differences among groups. (H) Quantification of Sertoli cells per stage VII–VIII seminiferous tubule confirming no change between UC-T, VC-T, and T males (n = 3 mice per condition, one tubule per mouse). Bars represent mean ± SD. Sertoli cell numbers were analyzed by one-way ANOVA. *Fshr* expression did not pass normality and was analyzed using the Kruskal–Wallis test (ns = not significant). Scale bar, 100 µm.

Tanis, Simon, et al., Supplemental Figure 7

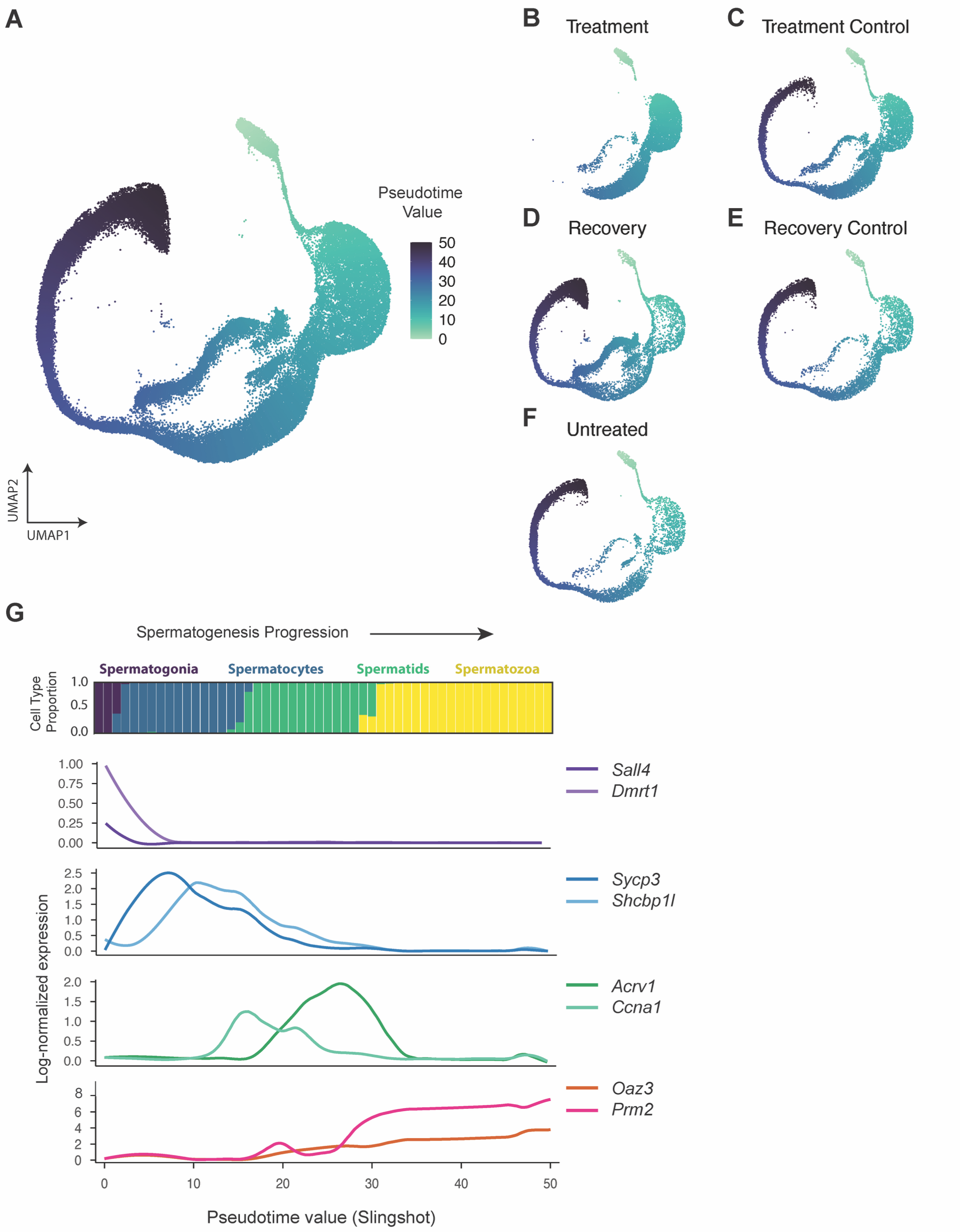

Figure S7. Pseudotime trajectory via Slingshot confirms ordered progression of spermatogenesis. (A) UMAP of germ cells colored by inverted Slingshot pseudotime value, illustrating a continuous trajectory. (B-F) Condition-specific UMAPs colored by pseudotime, showing consistent developmental ordering across Treatment (T) (B), Treatment Control (VC-T) (C), Recovery (R) (D), Recovery Control (VC-R) (E), and Untreated (UC) (F) groups. (G) Smoothed expression profiles of representative stage-specific genes along the Slingshot pseudotime. Early-stage factors (*Sall4, Dmrt1*) decline as meiotic genes (*Sycp3, Shcbp1l*) peak, followed by induction of post-meiotic transcripts (*Acrv1, Ccna1, Oaz3, Prm2*). The sequential activation of these gene modules confirms accurate reconstruction of spermatogenic progression.

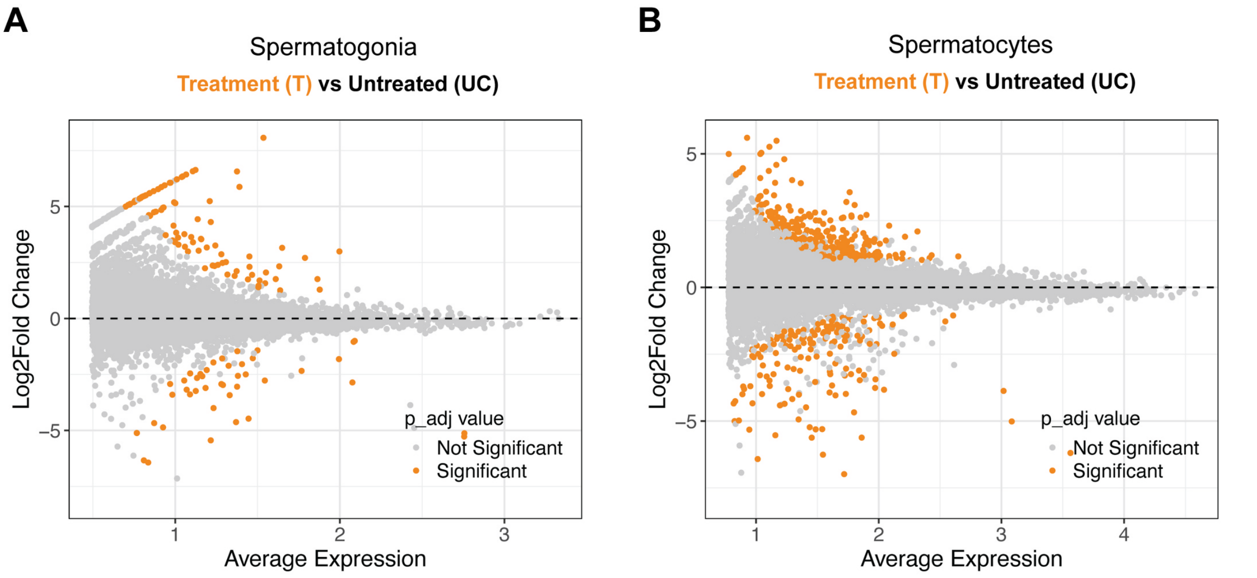

Figure S8. JQ1 induces modest transcriptional changes in early germ cells.

(A-B) MA plots showing differential expression between untreated (UC) and JQ1-treated (T) testes for (A) spermatogonia and (B) spermatocytes. Each point represents a gene, with significant differentially expressed (DE) genes shown in orange (adjusted $p < 0.05$ and $|\log_2 \text{fold change}| > 1$). JQ1 treatment altered 132 genes in spermatogonia and 372 genes in spermatocytes, with both upregulated and downregulated transcripts distributed around a $\log_2$ fold-change of zero.

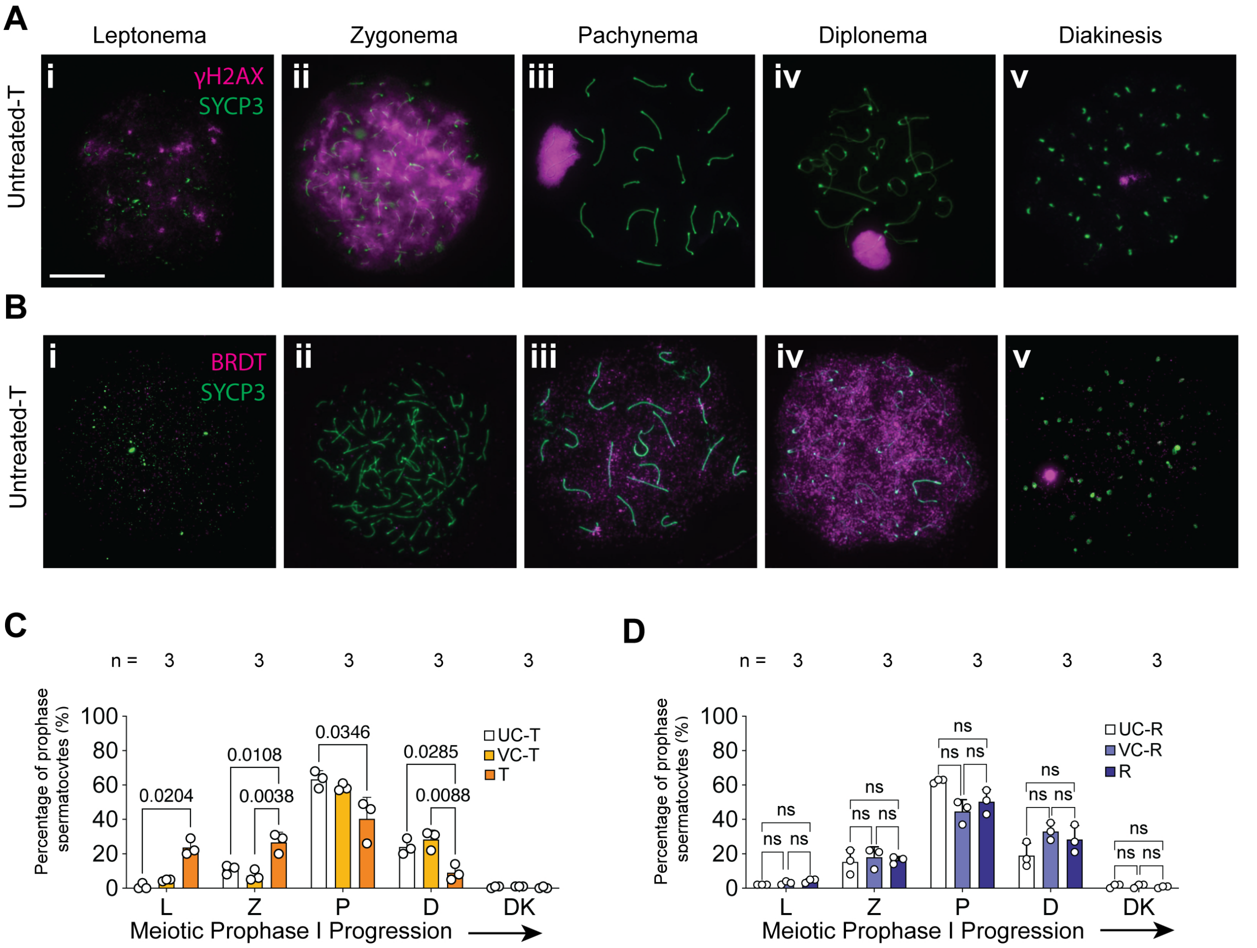

Figure S9. Meiotic staging and BRDT localization across prophase I in treatment and recovery cohorts. (A) Representative γH2AX (magenta) and SYCP3 (green) immunostaining across the five substages of meiotic prophase I - leptonema (L), zygonema (Z), pachynema (P), diplonema (D), and diakinesis (DK) - used to define staging criteria. (B) BRDT (magenta) and SYCP3 (green) staining across the same substages in UC-T testes, showing BRDT induction beginning at pachynema and persisting through diplonema. (C–D) Quantification of prophase I spermatocyte stage distribution in treatment cohorts (C; UC-T, VC-T, T) and recovery cohorts (D; UC-R, VC-R, R). Bars show mean ± SD; each point represents one biological replicate. Data were analyzed by the Kruskal–Wallis test followed by Dunn's multiple-comparison test (ns = not significant). Scale bars, 20 μm.

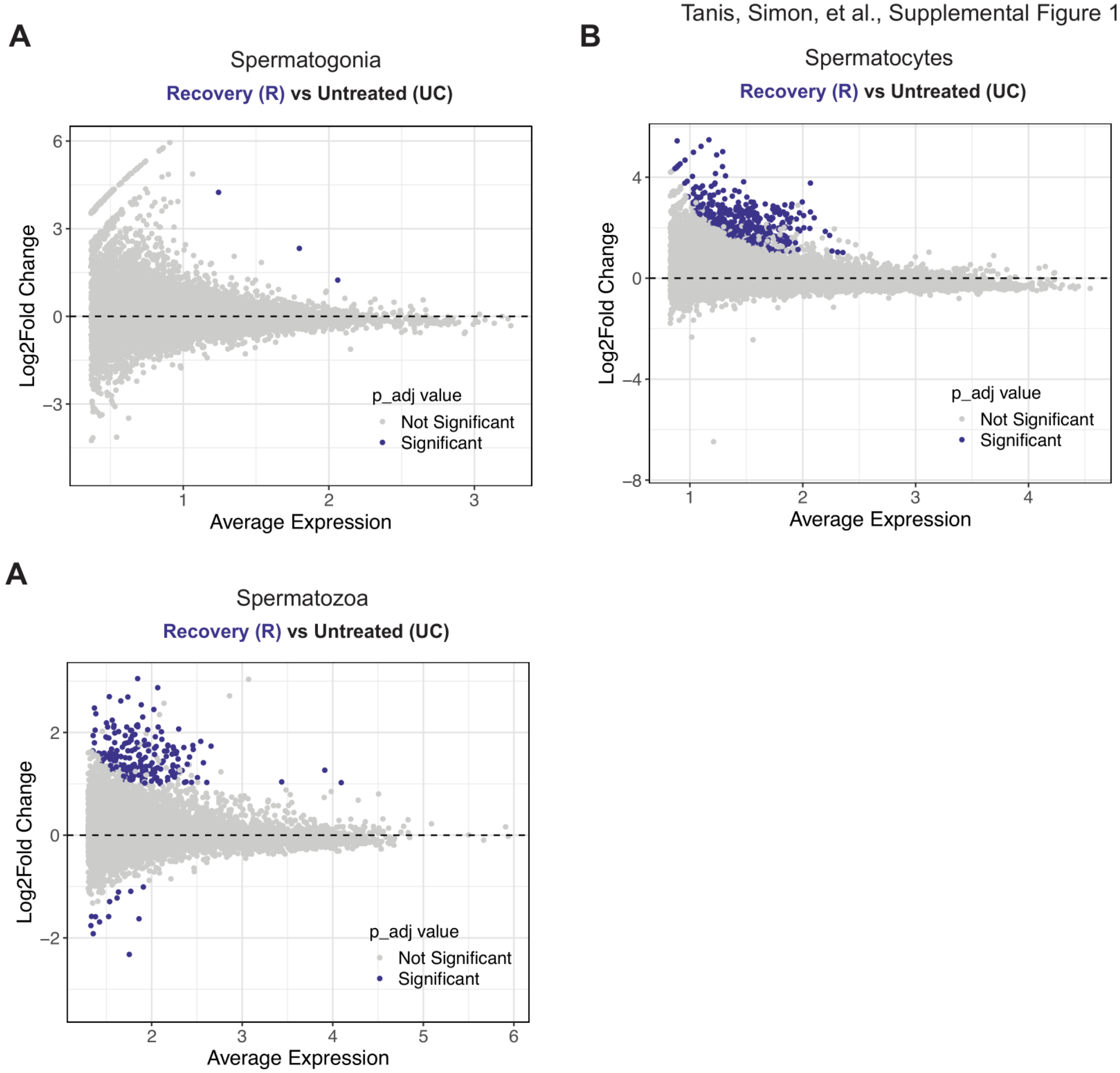

Figure S10. Recovery normalizes transcription across germ cell stages following JQ1 withdrawal. (A–C) MA plots showing differential expression between recovery (R) and untreated (UC) testes for (A) spermatogonia, (B) spermatocytes, and (C) spermatozoa. Each point represents a gene, with significantly differentially expressed (DE) genes shown in blue (adjusted $p < 0.05$ and $|\log_2 \text{fold change}| > 1$). Only 3 genes were dysregulated in spermatogonia, compared to 306 in spermatocytes and 130 in spermatozoa, demonstrating broad restoration of transcriptional homeostasis.

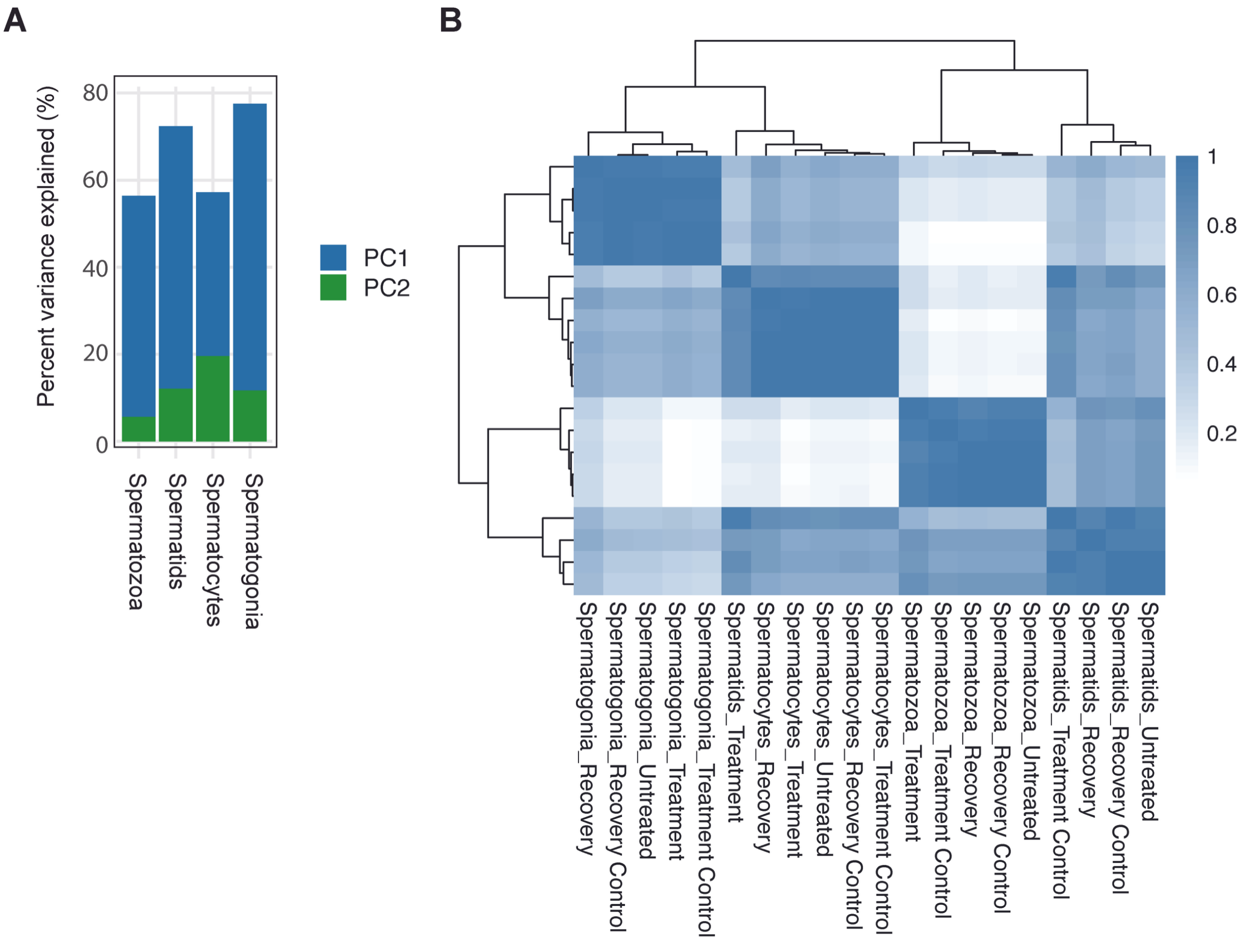

Figure S11. Quantitative validation of transcriptional recovery and inter-stage relationships. (A) Percentage of variance explained by the first two principal components (PC1 and PC2) for each germ-cell stage, showing that most transcriptional variation within each subset is captured by the top components. Spermatogonia: 56.42%; Spermatocytes: 72.4%; Spermatids: 57.22%; Spermatozoa: 77.57% (B) Pearson correlation heatmap of average gene expression profiles across germ-cell stages and experimental conditions. Samples cluster primarily by developmental stage, with strong intra-stage concordance between recovery (R), vehicle (VC-T, VC-R), and untreated (UC) groups. Notably, spermatids from JQ1-treated (T) testes cluster more closely with spermatocytes from other conditions than with spermatids, reflecting a treatment-induced transcriptional regression toward an earlier meiotic state. This shift is fully resolved after withdrawal, as recovery (R) spermatids realign with their stage-matched counterparts (VC-R, UC).

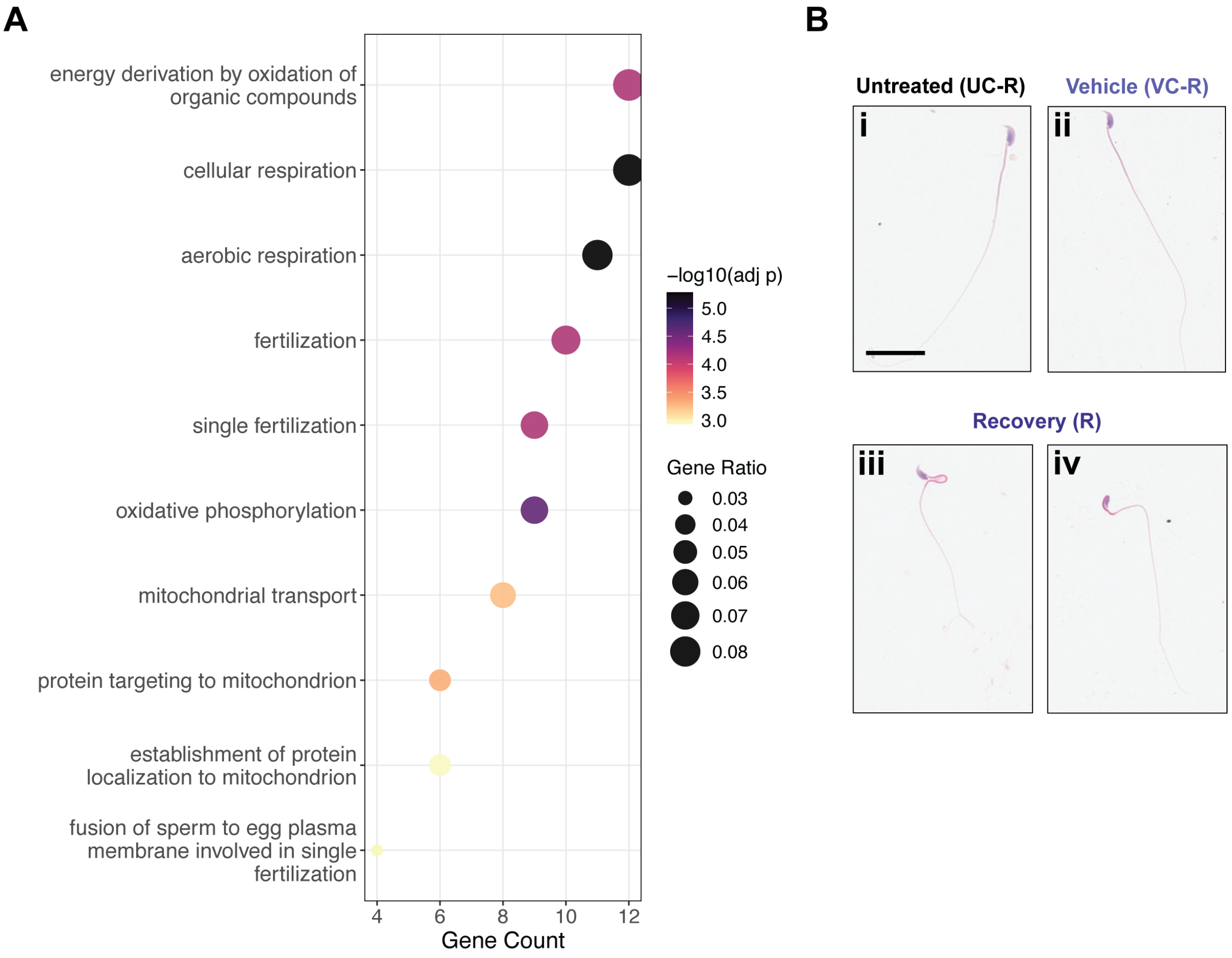

Figure S12. Expanded functional enrichment and morphology validation following recovery. (A) Gene Ontology (GO) enrichment analysis of genes that remained differentially expressed after recovery (spermatozoa, R vs UC). This panel presents the full set of significantly enriched biological processes corresponding to the summarized terms shown in Figure 3M, which highlights the five most representative categories. Circle size represents the gene ratio within each term, and color indicates statistical significance (–$\log_{10}$[adjusted p]). (B) Representative sperm morphology from untreated (i), vehicle (ii), and recovery (iii–iv) males. Recovery sperm largely regained normal head and tail architecture, though occasional abnormalities such as bent or kinked flagella persisted. Scale bar, 10 μm.

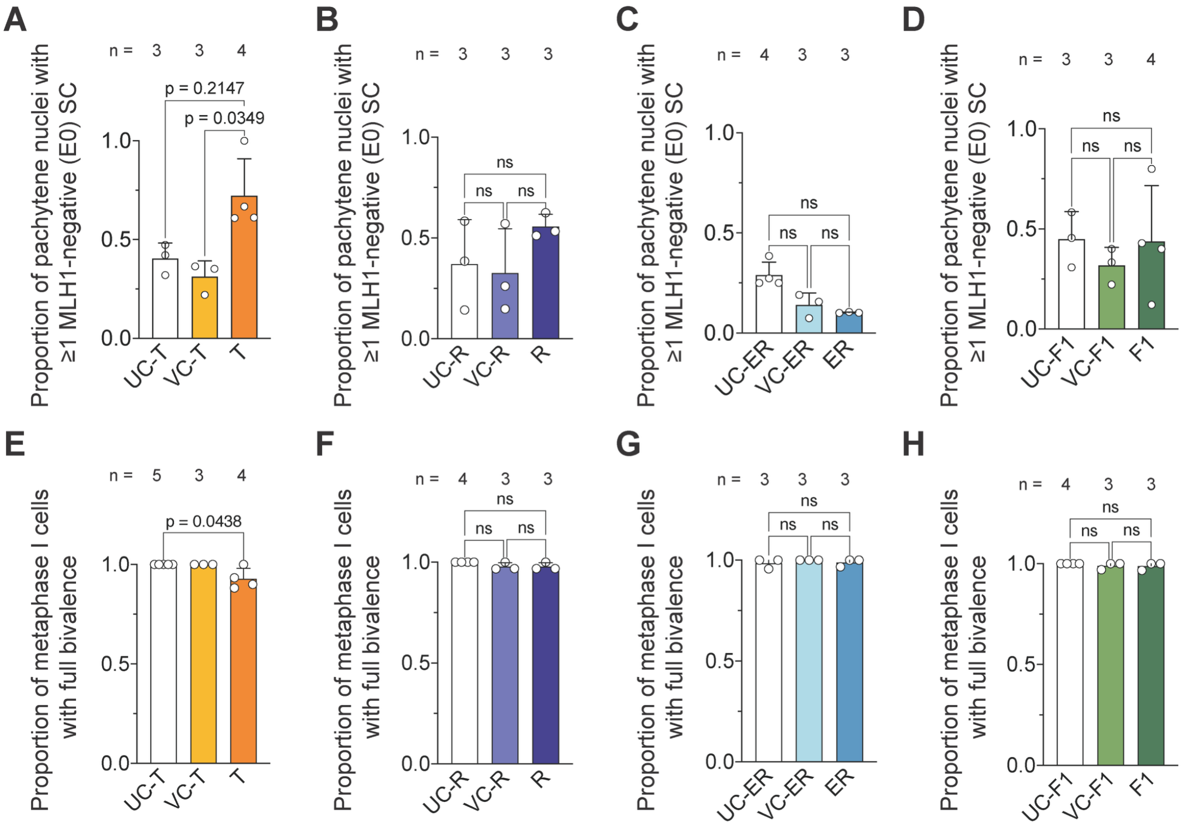

Figure S13. Proportion of pachytene nuclei with ≥1 MLH1-negative SC and metaphase I cells with full bivalence across treatment and recovery cohorts. (A–D) Proportion of pachytene nuclei with ≥1 MLH1-negative synaptonemal complex (SC) across treatment (UC-T,VC-T, T) (A), recovery (UC-R, VC-R, R) (B), extended recovery (UC-ER, VC-ER, ER) (C), and F1 (UC-F1, VC-F1, F1) (D) cohorts. (E–H) Proportion of metaphase I cells with full bivalence across the same timepoints. Bars represent mean ± SD; each point represents one biological replicate. All comparisons were performed using the Kruskal–Wallis test (ns = not significant).

Table S1. Metadata for datasets included in this study.

Sample Name	Condition	Number of reads	Reads mapped	Sequencing saturation	Estimated cell # prior to filtering	Estimated cell # after filtering	Cell # after Doublet Removal
JQ3WR1	Recovery	243,444,103	94.6%	32.0%	9,949	6,408	6,055
JQ3WR2	Recovery	275,053,459	90.4%	32.6%	10,785	7,191	6,792
JQ3WR3	Recovery	439,265,185	91.0%	34.7%	14,221	12,739	12,031
JQ3WRC1	Recovery Control	152,539,066	94.6%	28.1%	6,740	4,898	4,634
JQ3WRC2	Recovery Control	170,172,586	96.0%	35.2%	5,218	3,592	3,401
JQ3WRC3	Recovery Control	97,538,675	96.4%	31.3%	3,407	2,836	2,684
JQ3WT1	Treatment	175,844,767	95.2%	28.8%	9,567	5,694	5,551
JQ3WT2	Treatment	161,894,445	95.2%	34.0%	4,787	3,974	3,763
JQ3WT3	Treatment	144,378,699	94.9%	32.5%	3,573	2,527	2,397
JQ3WTC1	Treatment Control	155,707,767	96.0%	30.5%	6,932	5,746	5,443
JQ3WTC2	Treatment Control	200,624,393	96.2%	36.5%	7,080	3,521	3,330
JQ3WTC3	Treatment Control	167,287,369	96.0%	34.5%	5,042	3,595	3,405
UC1	Untreated	149,774,138	96.1%	34.3%	4,149	3,160	2,993
UC2	Untreated	154,707,378	92.9%	38.3%	4,071	2,975	2,817
UC3	Untreated	167,822,360	96.2%	35.0%	6,089	4,191	3,966
				Total:		**69,292**	

Table S2. Genes with BRDT-bound promoters differentially expressed in pachytene spermatocytes following JQ1 treatment. This table lists 16 genes identified as both BRDT promoter-occupied (based on BRDT ChIP-seq data from Her et al., (4)) and differentially expressed in pachytene spermatocytes from JQ1-treated testes. These overlapping genes represent putative direct BRDT transcriptional targets during meiotic prophase I. Gene names were harmonized to MGI-approved symbols, with alternative aliases noted (e.g., *Fopnl = 0610037P05Rik*).

#	Stage	Gene
1	Spermatocyte	*Akap1*
2	Spermatocyte	*Abhd2*
3	Spermatocyte	*Bclaf3*
4	Spermatocyte	*Ccnb3*
5	Spermatocyte	*D11Wsu47e*
6	Spermatocyte	*Dazl*
7	Spermatocyte	*Fmr1*
8	Spermatocyte	*Frat2*
9	Spermatocyte	*Hspa1l*
10	Spermatocyte	*Pgf*
11	Spermatocyte	*Prdx4*
12	Spermatocyte	*Spem1*
13	Spermatocyte	*Tsga8*
14	Spermatocyte	*Zfp768*
15	Spermatocyte	*Ubr7*
16	Spermatocyte	*Fopnl (0610037P05Rik)*

Table S3. Genes with BRDT-bound promoters differentially expressed in spermatids following JQ1 treatment. This table lists 46 genes identified as both BRDT promoter-occupied (based on BRDT ChIP-seq data from Her et al., (Her et al. 2021)) and differentially expressed in spermatids from JQ1-treated testes. These overlapping genes represent candidate direct BRDT transcriptional targets in late spermatogenesis. Gene symbols are based on Ensembl and MGI annotations, with aliases unified where appropriate (e.g., Tex35 = 1700057K13Rik).

#	Stage	Gene
1	Spermatid	*1700016C15Rik*
2	Spermatid	*1700065I17Rik (Tex43)*
3	Spermatid	*1700120B22Rik*
4	Spermatid	*1700023F06Rik*
5	Spermatid	*Actl9*
6	Spermatid	*Arf5*
7	Spermatid	*Akap4*
8	Spermatid	*Btg1*
9	Spermatid	*Cdc34*
10	Spermatid	*Cd109*
11	Spermatid	*Cldnd2 (Cldn25)*
12	Spermatid	*Dusp3*
13	Spermatid	*Eif4e*
14	Spermatid	*Eif4a2*
15	Spermatid	*Efcab3*
16	Spermatid	*Fopnl (0610037P05Rik)*
17	Spermatid	*Gfer*
18	Spermatid	*Glrx2*
19	Spermatid	*Habp4*
20	Spermatid	*Hdac11*
21	Spermatid	*Herpud1*
22	Spermatid	*Herpud2*
23	Spermatid	*Hspa1l*
24	Spermatid	*Isca1*
25	Spermatid	*Lrrc26*
26	Spermatid	*Mif*
27	Spermatid	*Ndufa13*
28	Spermatid	*Pfkfb2*
29	Spermatid	*Ppp1r15b*

30	Spermatid	*Prdx4*
31	Spermatid	*Rpf2*
32	Spermatid	*Rsl1d1*
33	Spermatid	*Snn*
34	Spermatid	*Spata3*
35	Spermatid	*Spem1*
36	Spermatid	*Tex35 (1700057K13Rik)*
37	Spermatid	Tnp1
38	Spermatid	Tnp2
39	Spermatid	Trpm8
40	Spermatid	Tubg1 (Tubb4b)
41	Spermatid	Ubr7
42	Spermatid	Uhrf1
43	Spermatid	Yipf6
44	Spermatid	Yod1
45	Spermatid	Zfp219
46	Spermatid	Zfp706

References

1. Chen S, Zhou Y, Chen Y, Gu J. fastp: an ultra-fast all-in-one FASTQ preprocessor. Bioinformatics. 2018 Sep 1;34(17):i884–90.
2. Soneson C, Love MI, Robinson MD. Differential analyses for RNA-seq: transcript-level estimates improve gene-level inferences. [version 2; peer review: 2 approved]. F1000Res. 2015 Jan 1;4:1521.
3. Love MI, Huber W, Anders S. Moderated estimation of fold change and dispersion for RNA-seq data with DESeq2. Genome Biol. 2014;15(12):550.
4. Her YR, Wang L, Chepelev I, Manterola M, Berkovits B, Cui K, et al. Genome-wide chromatin occupancy of BRDT and gene expression analysis suggest transcriptional partners and specific epigenetic landscapes that regulate gene expression during spermatogenesis. Mol Reprod Dev. 2021 Feb;88(2):141–57.
